# Clonal Clusters, Molecular Resistance Mechanisms and Virulence Factors of Gram-Negative Bacteria Isolated from Chronic Wounds in Ghana

**DOI:** 10.3390/antibiotics10030339

**Published:** 2021-03-22

**Authors:** Denise Dekker, Frederik Pankok, Thorsten Thye, Stefan Taudien, Kwabena Oppong, Charity Wiafe Akenten, Maike Lamshöft, Anna Jaeger, Martin Kaase, Simone Scheithauer, Konstantin Tanida, Hagen Frickmann, Jürgen May, Ulrike Loderstädt

**Affiliations:** 1 Department Infectious Disease Epidemiology, Bernhard Nocht Institute for Tropical Medicine Hamburg, 20359 Hamburg, Germany; thye@bnitm.de (T.T.); lamshoeft@bnitm.de (M.L.); anna.jaeger@bnitm.de (A.J.); may@bnitm.de (J.M.); 2 German Center for Infection Research (DZIF), Partner Site Hamburg-Lübeck-Borstel-Riems, 38124 Braunschweig, Germany; 3 Institute for Infection Control and Infectious Diseases, University Medical Center Göttingen, 37075 Göttingen, Germany; frederik.pankok@med.uni-goettingen.de (F.P.); stefan.taudien@med.uni-goettingen.de (S.T.); martin.kaase@med.uni-goettingen.de (M.K.); simone.scheithauer@med.uni-goettingen.de (S.S.); ulrike.loderstaedt1@med.uni-goettingen.de (U.L.); 4 Kumasi Centre for Collaborative Research in Tropical Medicine (KCCR), South-End, Asuogya Road, Kumasi 039-5028, Ghana; oppong@kccr.de (K.O.); danquah@kccr.de (C.W.A.); 5 Department of Microbiology and Hospital Hygiene, Bundeswehr Hospital Hamburg, External Site at the Bernhard Nocht Institute for Tropical Medicine Hamburg, 20359 Hamburg, Germany; konstantintanida@bundeswehr.org (K.T.); frickmann@bnitm.de (H.F.); 6 Institute for Medical Microbiology, Virology and Hygiene, University Medicine Rostock, 18057 Rostock, Germany; 7 Universitiy Medical Center Hamburg-Eppendorf (UKE), Tropical Medicine II, 20251 Hamburg, Germany

**Keywords:** wounds, Gram-negative bacteria, colonization, infection, clonal lineages, resistance genes, virulence factors

## Abstract

Wound infections are common medical problems in sub-Saharan Africa but data on the molecular epidemiology are rare. Within this study we assessed the clonal lineages, resistance genes and virulence factors of Gram-negative bacteria isolated from Ghanaian patients with chronic wounds. From a previous study, 49 *Pseudomonas aeruginosa*, 21 *Klebsiella* *pneumoniae* complex members and 12 *Escherichia coli* were subjected to whole genome sequencing. Sequence analysis indicated high clonal diversity with only nine *P. aeruginosa* clusters comprising two strains each and one *E. coli* cluster comprising three strains with high phylogenetic relationship suggesting nosocomial transmission. Acquired beta-lactamase genes were observed in some isolates next to a broad spectrum of additional genetic resistance determinants. Phenotypical expression of extended-spectrum beta-lactamase activity in the Enterobacterales was associated with *bla_CTX-M-15_* genes, which are frequent in Ghana. Frequently recorded virulence genes comprised genes related to invasion and iron-uptake in *E. coli*, genes related to adherence, iron-uptake, secretion systems and antiphagocytosis in *P. aeruginosa* and genes related to adherence, biofilm formation, immune evasion, iron-uptake and secretion systems in *K. pneumonia* complex. In summary, the study provides a piece in the puzzle of the molecular epidemiology of Gram-negative bacteria in chronic wounds in rural Ghana.

## 1. Introduction

The microbiology of chronic infected wounds, also on a molecular level, is poorly understood in sub-Saharan Africa (SSA) [1]. However, studies highlight the importance of antibiotic resistant Gram-negative bacteria [2,3,4,5,6].

From other parts in the world, in particular from industrialized countries, information on the microbiology and the role of biofilm-forming microorganisms causing such infections are well established [7,8,9,10].

In chronic wounds, *Pseudomonas aeruginosa* is amongst the most frequently isolated Gram-negative bacteria, associated with biofilm formation [11,12]. Tightly adhering biofilms pose a challenge in the diagnosis of *P. aeruginosa* using standard culturing methods [13].

In comparison, the role of Enterobacterales in chronic wounds has been much less characterized [14,15,16,17]. Studies have shown that geography seems to play a role in the estimation of their etiological relevance [18]. It was shown that skin colonization with Gram-negative bacteria is frequent in resource-limited (sub)tropical settings [19,20,21], in contrast to skin colonization of individuals from industrialized countries, where Gram-positive bacteria dominate [19]. Temperature and moisture have been discussed as likely reasons for the difference seen [22].

Isolation of potentially pathogenic bacteria from non-sterile sites like wounds does not necessarily indicate clinical relevance, which poses challenge to clinical interpretation.

In a recent study that focused on the overall bacterial composition of chronic wound infections in Ghana, from which the isolates for the present molecular analysis were taken, Enterobacterales and *Pseudomonas aeruginosa* constituted the majority of isolated bacterial strains [23]. A moderate proportion of ESBL-positive Enterobacterales suggests lower frequencies of antibiotic resistance [23] than what was recorded from other Ghanaian hospitals [5,24].

Within this study, we aim at characterizing clonal lineages, resistance-associated genetic elements and virulence genes of *P. aeruginosa*, the *Klebsiella pneumoniae* complex and *Escherichia coli*, which were recently isolated from chronic wounds of Ghanaian adult patients [23]. The molecular epidemiology of dominating clonal lineages and associated resistance genes will be assessed. Further, analysis of highly abundant virulence factors will be conducted.

## 2. Results

### 2.1. Clustering Based on Core Genome Multilocus Sequence Typing (cgMLST) Results

**Figure 1:**
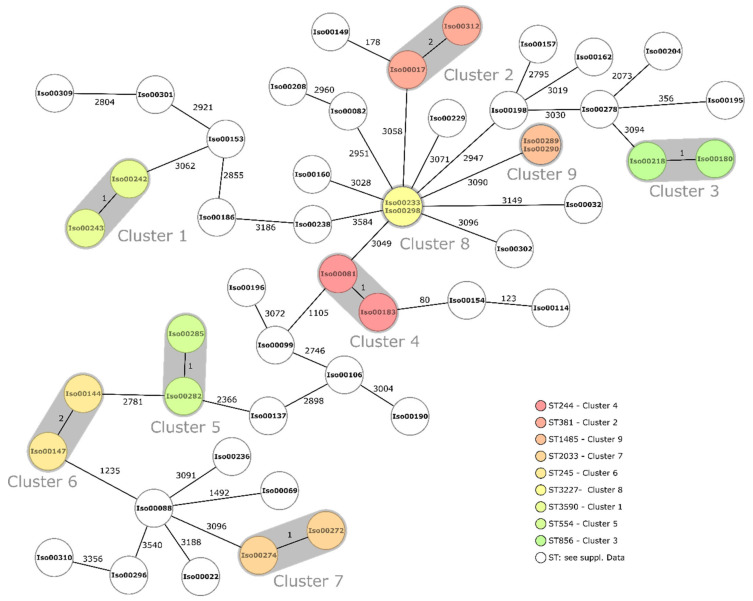
Minimum spanning tree of *P. aeruginosa* based on 3867 targets (core genome). Isolate numbers are found within the nodes, and numbers between nodes indicate the number of different alleles. Isolates within clusters are colored based on MLST sequence type (ST). The ST types of white nodes are indicated in Table A1.

Of the 49 *P. aeruginosa* analyzed, a total of nine clusters comprising isolates without any recorded differences (*n* = 2) or with one or two alleles difference (*n* = 7) were found, suggesting closely related phylogeny (Figure 1). In addition to the clusters, 31 singletons with differences ranging from 80 to 3584 alleles were observed. MLST sequence types (ST) are indicated in Figure 1 and Table A1 and Table A2. Cluster sequence types included the following: ST244, ST245, ST381, ST554, ST856, ST1485, ST2033, ST3227 and ST3590.

**Figure 2:**
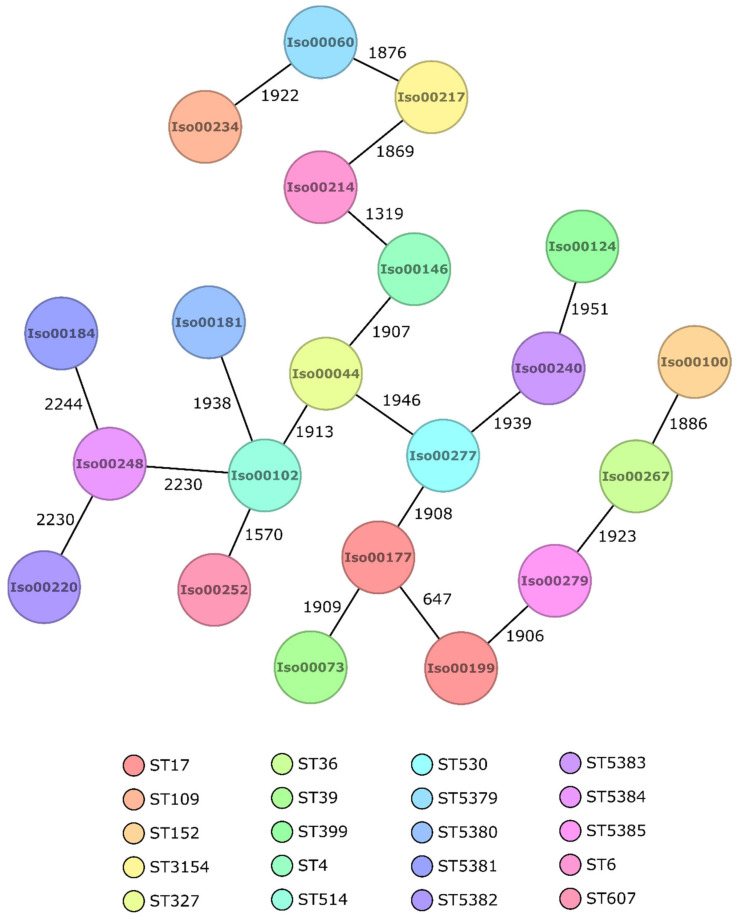
Minimum spanning tree of *K. pneumoniae* complex based on 2358 targets (core genome). Isolate numbers are found within the nodes, and the numbers between the nodes indicate the number of different alleles. Colors demonstrate the MLST sequence type of the isolates.

**Figure 3:**
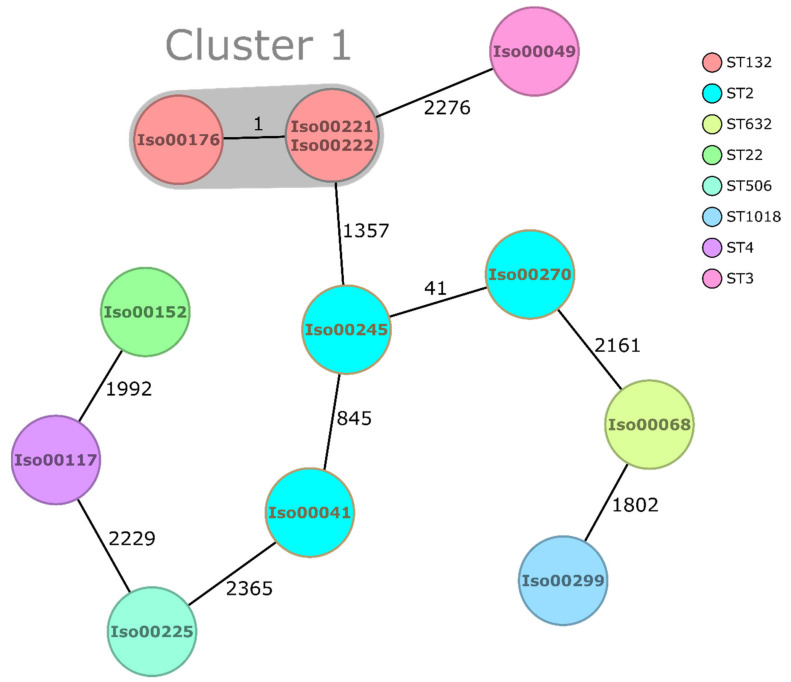
Minimum spanning tree of *E. coli* based on 2513 targets (core genome). Isolate numbers are found within the nodes, and the numbers between the nodes indicate the number of different alleles. Colors demonstrate the Pasteur sequence type of the isolates.

No clusters were identified among the 21 assessed *K. pneumonia* complex members, which were all singletons with differences ranging from 647 to 2244 alleles. *K. pneumoniae* complex sequence types are summarized in Figure 2. From the 12 *E. coli* isolates, three isolates in a cluster of close phylogenetic relationship were found (1× no allelic differences, 1 × 1 allele difference) (Figure 3). In addition to the cluster observed, nine singletons with differences ranging from 41 to 2365 alleles were recorded. The sequence type of the cluster was ST132 (Pasteur MLST scheme). Sequence types of all *E. coli* isolates are illustrated in Figure 3.

### 2.2. Identified Molecular Resistance Mechanisms in Correlation to Previous Phenotypic Antibiotic Resistance 

Table 1 summarizes acquired antimicrobial resistance determinants for *E. coli* and acquired genes mediating tolerance to disinfectants. Data for *P. aeruginosa* and *K. pneumoniae* are presented in Table A1 and Table A2. Table A3, Table A4, Table A5, Table A6, Table A7 and Table A8 summarize the phenotypic resistance results as previously recorded [23].

In the present study, phylogenetically identical or almost identical isolates also carried the same resistomes. All *E. coli* strains harbored acquired beta-lactamase genes with the majority coding for small spectrum beta-lactamases such as *bla*_TEM-1_ or *bla*_OXA-1_. Only four strains carried the gene for an ESBL, in all cases *bla*_CTX-M-15_. Among the *K. pneumoniae* complex strains, two belonged to the species *K. variicola*, one to the species *K. quasipneumoniae* and the remaining to the species *K. pneumoniae* sensu stricto as reflected by intrinsic *bla*_LEN_, *bla*_OKP_ and *bla*_SHV-1 like_, respectively. Genes coding for ESBL (*bla*_CTX-M-15_) were found solely in four out of 18 *K. pneumoniae* sensu stricto strains that also displayed resistance to oxyimino cephalosporins. In addition, several *K. pneumoniae* complex strains harbored *bla*_TEM-1_, single strains also contained *bla*_OXA-1_ and *bla*_SCO-1_.

**Figure A1:**
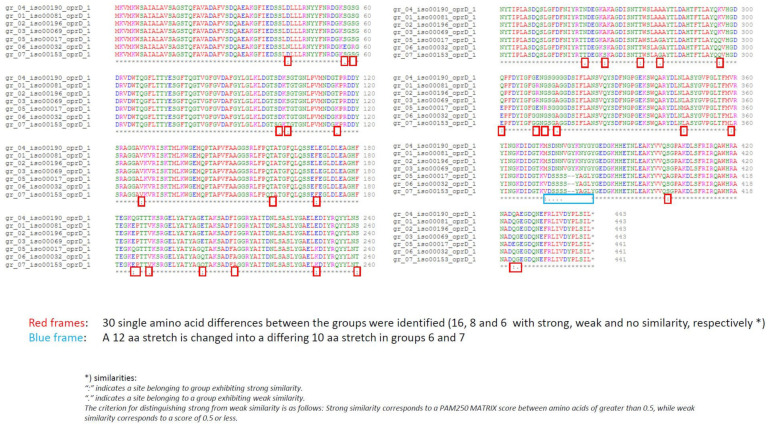
Clustal omega multiple alignment of oprD proteins—one example for the 7 detected subgroups.

With respect to *P. aeruginosa*, only one strain harbored acquired beta-lactamase genes (*bla*_TEM-1_ and *bla*_SCO-1_). Increased minimum inhibitory concentrations (MICs) for carbapenems as observed in some *P. aeruginosa* strains were neither explained by matching acquired carbapenemase genes nor by full sequence analysis of the *oprD* gene. The associated amino acid sequences are shown in Figure A1. As indicated, the complete *oprD* gene was found in all 49 *P. aeruginosa* isolates; there was no evidence of protein truncation by premature stop of translation. The 49 isolates could be divided into 7 subgroups according to the protein sequence of the oprD protein, which differ in a total of 30 individual amino acid exchanges and in a single 12aa/10aa-stretch. Therefore, genotypic assessment could not identify the reason for the single carbapenem-resistant *P. aeuroginosa* isolate 088 (ST 1682).

Other frequently detected resistance genes in *P. aeruginosa* were the fosfomycin resistance gene *fosA*, the chloramphenicol resistance gene *catB7*, the aminoglycoside resistance gene *aph(3′)-IIb* and the fluoroquinolone-resistance gene *crpP*. In the *Klebsiella pneumoniae* complex isolates, single amino acid exchanges and the fosmomycin resistance gene *fosA* were frequent. Various fluoroquinolone resistance genes and disinfectant tolerance mediating genes also quantitatively dominated. Finally, a broad spectrum of acquired genes causing resistance to the assessed classes of antimicrobial drugs and tolerance to disinfectants was observed in the *E. coli* strains.

### 2.3. Identified Molecular Virulence Mechanisms

Table 2 summarizes the analysis of virulence-related genes in *E. coli* (without genes mediating enteropathogenicity). Data for *P. aeruginosa* and *K. pneumoniae* are presented in Table A9 and Table A10.

The virulence-associated gene *exoU*, which has been described in association with the *P. aeruginosa* high-risk clone ST 135 [25], was recorded three times, associated with ST 135 (sample ID 296), ST 532 (sample ID 310) and ST 2483 (sample ID 22), respectively. Based on a Kleborate assessment, a positive virulence score was calculated for 7 out of 21 *K. pneumoniae* strains, comprising the known high-risk clones ST 17 (sample IDs 177, 199) and ST 152 (sample ID 100) [26], next to the clones ST 4 (sample ID 146), ST 6 (sample ID 214), ST 36 (sample ID 267) and ST 39 (sample ID 73), respectively. With focus on some important virulence associated genes in *Klebsiella* spp., *ybt* genes were detected in the abovementioned 7 samples, *iroE* was recorded in all 21 strains, while *clb* or *rpmA* genes were not detected.

Iron-uptake-related genes were numerous in all analyzed bacterial strains. For *P. aeruginosa* and *K. pneumoniae*, various secretion system-associated genes were found. Immune evasion-related genes were highly abundant in *K. pneumoniae* but not in *E. coli* isolates. Adherence-related genes were numerous in *P. aeruginosa* and in *K. pneumoniae* but not in *E. coli*.

Numerous invasion-associated genes were detected in *E. coli*, antiphagocytosis-associated genes were found in *P. aeruginosa*, and biofilm-associated genes in *K. pneumoniae*.

Less frequently detected were: toxin genes in *E. coli* and *K. pneumoniae*, protease genes in *E. coli* and *P. aeruginosa*, regulation genes in *P. aeruginosa* and *K. pneumoniae*, biosurfactant and pigment genes in *P. aeruginosa* and nutrition factor, efflux pumps and serum resistance genes in *K. pneumoniae*.

## 3. Discussion

Within this study, we aimed at filling information gaps on the molecular epidemiology of Gram-negative bacteria from chronic infected wounds in rural Ghana. Phylogenetic analyses based on core genome comparison indicated a high clonal diversity of the wound-associated isolates. Clonal clusters were restricted to nine *P. aeruginosa* clusters and one *E. coli* cluster, most likely indicating nosocomial transmission, which has most likely occurred in the wound dressing room that patients’ visit on a weekly basis.

ST 135 and ST 244, which are among the worldwide top 10 *P. aeruginosa* high-risk clones [25], were found among the *P. aeruginosa* wound isolates. In detail, one ST 135 *Pseudomonas aeruginosa* isolate was detected, carrying the beta-lactamase-encoding genes *bla*_TEM-1B_ and *bla*_SCO-1_ and an *exoU* gene, next to five ST 244 without acquired beta-lactamases. Focusing on known pathogenic *K. pneumoniae* clones [26], two ST 17 strains, a clone reported to be associated with carbapenem-resistance, and one ST 152 strain, a clone known from the Caribbean as common carrier of multiple resistance genes, were detected. Strains carrying the *ybt* and *iro* genes were also identified as high-risk clones by the Kleborate software. From the observed *E. coli* ST types, none have been previously reported as being associated with pathogenic clones so far [27].

In line with the phenotypical antibiotic resistance results previously published [23], numerous acquired resistance determinants were detected in the bacterial strains under investigation. Focusing on the few observed clusters, comparable resistome compositions point towards recent nosocomial transmission. The gene *bla*_CTX-M-15_ was identified as the determinant of the detected extended spectrum beta-lactamase (ESBL) expression in ESBL positive Enterobacterales [23]. This is in line with previous reports from both human and livestock-associated ESBL positive Enterobacterales in Ghana [28,29,30,31,32,33,34]. In *P. aeruginosa* and *K. pneumoniae*, *bla*_SCO-1_, which has initially been described from an *Acinetobacter baumannii* isolate from Argentina [35], was observed. Beta-lactamases with high hydrolytic effects on carbapenems were lacking, the same applies to protein truncation by premature stop of translation of the *oprD* gene in *P. aeruginosa*. Accordingly, the genetic background of carbapenem resistance of a single *P. aeruginosa* strain could not be resolved, although downregulation of *oprD* expression due to mutations outside of the gene or *amp*C (class C betalactamase) overexpression could not be excluded as likely reasons.

Substance-specific genes and genes encoding efflux pumps mediating tolerance to disinfectants were observed in Enterobacterales. Therefore, further monitoring of the spread of disinfectant tolerance-associated genes and the effects of their abundance on disinfectant-based skin and wound decolonization strategies [36] seem advisable.

The importance of highly abundant virulence factors like iron-uptake- and secretion system-related genes in *P. aeruginosa* is comprehensively described in the literature [37,38]. Other genes reported in the literature like regulation-associated virulence genes, recently reported, were less frequently observed in our isolates [39,40]. However, due to lacking information on the individual etiological relevance of each isolate, any association with clinical effects remains speculative.

Further limitations of this study include a rather small sample size and the lack of a comparison strain collection containing isolates from other clinical specimens and environmental strains. Accordingly, the interpretation of the etiological relevance of individual strains remains challenging and is clearly beyond the scope of this work.

In summary, a broad spectrum of Gram-negative clones was isolated from the chronic wounds of the Ghanaian patients. Thereby, known high-risk clones [25,26,27] played only a minor role. Observed resistance patterns and mechanisms were in line with the spectrum expected from previous reports [23,28,29,30,31,32,33,34].

## 4. Materials and Methods

### 4.1. Sample Collection, Bacterial Culture and Antibiotic Susceptibility Testing

Single patient strains of *P. aeruginosa, E. coli* and *K. pneumoniae* complex were isolated from patients ≥15 years with an infected chronic wound at the Outpatient Department (OPD) of the Agogo Presbyterian Hospital, in the Asante Akim North District of rural Ghana. Patients typically visit the wound dressing room of the OPD on a weekly basis. Sampling was performed from January 2016 to November 2016. Sample collection and microbiological investigations were reported previously [23]. Antibiotic susceptibility was tested by the disk diffusion method and interpreted following the European Committee on Antimicrobial Susceptibility Testing (EUCAST) guidelines v.6.0 (http://www.eucast.org (accessed on 15 January 2016)). Bacterial strains and antibiotic susceptibility were confirmed using the VITEK2 System. Those data have been published before [23].

### 4.2. DNA Isolation and Whole Genome Sequencing

Bacterial DNA was isolated using the MasterPure Complete DNA and RNA Purification Kit (LGC standards GmbH, Wesel, Germany) and sent for whole genome sequencing (WGS) to BGI Europe, Denmark, Copenhagen. A BGISEQ-500 device was used for sequencing, generating 2 × 150 bp paired-end reads with an aimed coverage of 100×. Original raw data were upload for public use to the short-read archive (SRA, NCBI) under the accession number PRJNA699140. Details on the strain-specific SRA accession numbers are provided in Table A11.

### 4.3. Whole Genome Sequencing and Data Analysis

All raw data passed quality control using FASTQC v.0.11.4 [41] and were used for further analysis. Taxonomic classification and contamination check of raw-reads was performed using KRAKEN2 v.2.0.8-beta [42]. Phylogenetic analysis based on core genome multi locus sequence typing (cgMLST) analysis was performed using the commercial software SeqSphere+ v. 7.2.0 (Ridom GmbH, Münster, Germany) [43]. The software pipeline included assessment of read data and adapter control using FASTQC followed by genome assembly using the internally provided assembler Velvet, applying default settings. The reference genomes NC_000913.3 (*E. coli*), NC_002516.2 (*P. aeruginosa*) and NC_01273.1 (*K. pneumoniae* species complex) were used for cgMLST analyses. Only samples with a ration of “good cgMLST targets” higher than 90% were included in the phylogenetic analysis. Novel cgMLST-based complex types (CT) were automatically assigned by the SeqSphere software. Unknown alleles and profiles of MLST genes were submitted to pubmlst.org or Institute Pasteur to establish novel sequence types (ST). Isolates were defined to be clonally identical with allele differences less than four. Moreover, raw data were assembled with SPAdes v3.13.11 [44] using the careful option. Scaffolds shorter than 500 bp or with a coverage smaller than ten were sorted out, using an in-house script. Abricate v.0.9.9 [45] was used to screen for resistance and virulence genes in SPAdes assembly files, using NCBI AMRFinderPlus [46] and VFDB [47] as reference databases (both updated 6 November 2020), respectively. Additionally, SPAdes assemblies were uploaded to ResFinder4.1 [48] to obtain WGS predicted phenotypes against different antimicrobials by using default settings (%ID > 90, minimum length > 60%) and to Kleborate to predict virulence genes in *Klebsiella* isolates.

### 4.4. Ethical Considerations

The Committee on Human Research, Publications and Ethics, School of Medical Science, Kwame Nkrumah University of Science and Technology in Kumasi, Ghana, approved this study (approval number CHRPE/AP/078/16).

## 5. Conclusions

In conclusion, this study provides a molecular insight into the epidemiology of Gram-negative bacteria isolated from chronic wound infections from patients in rural Ghana. Epidemiological data that focus on the distribution and spread of antimicrobial resistance determinants and associated virulence factors in resource-limited settings are scarce. Although the study is a small cross-sectional assessment, which cannot replace continuous surveillance programs, it might provide a glimpse of prevailing Gram-negative bacteria isolated from wound infections in this area of Ghana. Considering the ongoing need for resistance and virulence surveillance in tropical regions, larger future studies are desirable.

## Figures and Tables

**Table 1:** Analysis of antimicrobial resistance determinants, ordered by strain and MLST type, of the assessed *E. coli* isolates. ST = Sequence type.

Sample ID	ST-Type	Acquired Resistance Determinants Against										
		Beta lacatams	Sulfonamids	Trimethoprim	Makrodlids	Tetracyclins	Fluoroquinolones	Chloramphenicol	Aminoglycosides	Efflux pumps	Amino acid exchanges due to point mutations	Disinfectant resistance genes *
041	ST 2	*bla*_OXA-1_, *bla*_TEM-1B_, *bla*_CTX-M-15_	*sul1*	*dfrA17*	*mph(A)*	*tet(B)*	*aac(6′)-Ib-cr, aac(6′)-Ib-cr*	*catB3,* *catA1*	*aac(3)-IId, aac(6′)-Ib-cr, aadA5, aac(6′)-Ib-cr*	*mdf(A)*	*parE* p.S458A, *gyrA* p.S83L, *gyrA* p.D87N, *parC* p.S80I	*sitABCD*, *qacE*
049	ST 3	*bla*_TEM-1B_	*sul2, sul1,*	*dfrA12*	*mph(A)*	*tet(A)*			*aadA2, aph(3″)-Ib, aph(6)-Id*	*mdf(A)*		*sitABCD*-like, *qacE*
068	ST 632	*bla*_TEM-1B_	*sul3*	*dfrA12*		*tet(A)*		*cmlA1*	*aadA1, aadA2*	*mdf(A)*-like	*parE* p.S458A, *gyrA* p.S83L, *gyrA* p.D87N, *parC* p.S80I	
117	ST 4	*bla*_TEM-1B_	*sul1, sul2*	*dfrA7*		*tet(A)*		*catA1*	*aph(6)-Id, aph(3* *″)-Ib*	*mdf(A)*-like		*sitABCD*-like, *qacE*
152	ST 22	*bla*_CARB-2_, *bla*_TEM-1B_	*sul1*	*dfrA1*	*ere(B)*	*tet(B)*		*catA1*	*aadA1, aadA2b*	*mdf(A)*-like	*gyrA* p.S83L	*qacE*, *sitABCD*
176	ST 132	*bla*_TEM-1B_	*sul1*	*dfrA7*		*tet(A)*		*catA1*	*aph(3* *″)-Ib, aph(6)-Id*	*mdf(A)*		*qacE*, *sitABCD*
221	ST 132	*bla*_TEM-1B_	*sul1*	*dfrA7*		*tet(A)*		*catA1*	*aph(6)-Id, aph(3* *″)-Ib*	*mdf(A)*		*qacE*, *sitABCD*
222	ST 132	*bla*_TEM-1B_	*sul1, sul2*	*dfrA7*		*tet(A)*		*catA1*	*aph(3* *″)-Ib, aph(6)-Id*	*mdf(A)*		*qacE*, *sitABCD*
225	ST 506	*bla*_TEM-1D_, *bla*_CTX-M-15_	*sul1, sul2*	*dfrA17*	*mph(A)*	*tet(A)*		*catA1*	*aadA5, aph(6)-Id, aph(3″)-Ib*	*mdf(A)*-like	*gyrA* p.S83L, *parE* p.I529L	*sitABCD*-like, *qacE*
245	ST 2	*bla*_TEM-1B_	*sul1*	*dfrA12*	*mph(A)*	*tet(B)*	*qepA4 (neu)*	*catA1*	*aadA2, aac(3)-IId*	*mdf(A)*	*parE* p.S458A, *gyrA* p.S83L, *gyrA* p.D87N, *parC* p.S80I	*qacE*
270	ST 2	*bla*_CTX-M-15_				*tet(B)*		*catA1*		*mdf(A)*	*gyrA* p.S83L, *gyrA* p.D87N, *parE* p.S458A, *parC* p.S80I	
299	ST 1018	*bla*_TEM-1B_	*sul3*	*dfrA14*		*tet(A)*	*qnrS1*			*mdf(A)*		

* *sitABCD* = peroxides resistance, *qacE* = quaternary ammonium compounds resistance.

**Table 2:** Analysis of virulence determinants, ordered by strain and MLST type, of the assessed *E. coli* isolates. ST = Sequence type.

Sample ID	ST-Type	Pathogenicity Factor Groups					
		Adherence	Invasion	Toxin	Immune Evasion	Iron Uptake	Protease
041	ST 2	*fdeC*	*aslA*, *ompA*			*entA*-like, *entB*, *entC*, *entE*, *entF*, *entS*, *fepA*, *fepB*, *fepC*, *fepD*, *fepG,*	
049	ST 3		*aslA, kpsC, kpsD, kpsE, kpsF, kpsM, kpsU, kpsS*-like, *ompA*			chuS, chuU, chuV, chuW, chuY, *entA*-like, *entB*, *entC*, *entE*, *entF*, *entS*, *fepA*, *fepB*, *fepC*, *fepD*, *fepG*	
068	ST 632		*ompA*			*entA*-like, *entB*, *entC*, *entE*, *entF*, *entS*, *fepA*, *fepB*, *fepC*, *fepD*, *fepG*	
117	ST 4		*aslA, kpsC, kpsD, kpsE, kpsF, kpsM, kpsU; kpsS*-like, *ompA*	*hlyB, hlyC, hlyD,*	*tcpC*	chuA, chuS, chuT, chuU, chuV, chuW, chuX, chuY, *entA*-like, *entB*, *entC*, *entE*, *entF*, *entS*, *fepA*, *fepB*, *fepC*, *fepD*, *fepG, hlyA, iroN,*	*pic, sat, vat*
152	ST 22	*sfaB, sfaC, sfaD, sfaE, sfaF, sfaG, sfaH, sfaS, sfaX, sfaY*	*aslA, kpsC, kpsD, kpsE, kpsF, kpsM, kpsU; kpsS*-like, *ompA*	*cnf1; hlyA, hlyB, hlyC, hlyD,*	*tcpC*	*chuA, chuS, chuT, chuU, chuV, chuW, chuX, chuY, entA-like, entB, entC, entE, entF, entS, fepA, fepB, fepC, fepD, fepG, iroN,*	*vat*
176	ST 132		*aslA, kpsC, kpsD, kpsE, kpsF, kpsM, kpsU; kpsS*-like, *ompA*			*entA-like, entB, entC, entE, entF, entS, fepA, fepB, fepC, fepD, fepG,*	*sat*
221	ST 132		*aslA, kpsC, kpsD, kpsE, kpsM, kpsU; kpsS*-like, *ompA*			*entA*-like, *entB, entC, entE, entF, entS, fepA, fepB, fepC, fepD, fepG,*	*sat*
222	ST 132		*aslA, kpsC, kpsD, kpsE, kpsF, kpsM, kpsU; kpsS*-like, *ompA*			*entA-like, entB, entC, entE, entF, entS, fepA, fepB, fepC, fepD, fepG,*	*sat*
225	ST 506		*aslA, kpsC, kpsD, kpsE, kpsF, kpsM, kpsU; kpsS*-like, *ompA*			*chuA, chuS, chuT, chuU, chuV, chuW, chuX, chuY, entA*-like, *entB, entC, entE, entF, entS, fepA, fepB, fepC, fepD, fepG,*	*sat*
245	ST 2		*aslA, ompA*			*entA-like, entB, entC, entE, entF, entS, fepB, fepC, fepD, fepG*	
270	ST 2		*aslA, ompA*			*entA-like, entB, entC, entE, entS, fepA, fepB, fepC, fepD, fepG*	
299	ST 1018		*ompA*			*entA*-like, *entB, entC, entE, entF, entS, fepA, fepB, fepD, fepG*	

## Data Availability

All relevant data have been provided in the paper and its Appendix A materials. Raw data are available applying the links as indicated in the methods chapter and can also be provided by the authors on reasonable request.

## Appendix A

**Table A1:** Analysis of antimicrobial resistance determinants, ordered by strain and MLST type, of the assessed *P. aeruginosa* isolates. ST = Sequence type.

Sample ID	ST-Type	Acquired Resistance Determinants Against												
		Beta Lacatams	Sulfonamids	Fosfomycin	Trimethoprim	Makrolides	Tetracyclinws	Fluoroquinolones	Chloramphenicol	Rifampicin	Aminoglycosides	Efflux Pumps	Amino Acid Exchanges Due to Point Mutations	Disinfectant Resistance Genes
017	ST 381			*fosA*					*catB7*		*aph(3′)-IIb*			
022	ST 2483			*fosA*					*catB7*		*aph(3′)-IIb*			
032	ST 3587		*sul1*	*fosA*	*dfrA15*		*tet(G)*		*catB7*		*aph(3′)-IIb*			
069	ST 360			*fosA*				*crpP*-like	*catB7*		*aph(3′)-IIb*			
081	ST 244			*fosA*					*catB7*		*aph(3′)-IIb*			
082	ST 514			*fosA*					*catB7*		*aph(3′)-IIb*			
088	ST 1682			*fosA*					*catB7*		*aph(3′)-IIb*			
099	ST 244			*fosA*					*catB7*		*aph(3′)-IIb*			
106	ST 1521			*fosA*					*catB7*		*aph(3′)-IIb*			
114	ST 244			*fosA*				*crpP*-like	*catB7*		*aph(3′)-IIb*-like			
137	ST 3014			*fosA*				*crpP*-like	*catB7*		*aph(3′)-IIb*-like			
144	ST 245			*fosA*				*crpP*-like	*catB7*		*aph(3′)-IIb*			
147	ST 245			*fosA*				*crpP*-like	*catB7*		*aph(3′)-IIb*			
149	ST 381			*fosA*				*crpP*-like	*catB7*		*aph(3′)-IIb*			
153	ST 704			*fosA*-like				*crpP*-like	*catB7*-like		*aph(3′)-IIb*- like			
154	ST 244	*,*		*fosA*				*crpP*-like	*catB7*		*aph(3′)-IIb*-like			
157	ST 2616			*fosA*					*catB7*-like		*aph(3′)-IIb*			
160	ST 170			*fosA*-like							*aph(3′)-IIb*			
162	ST 274			*fosA*				*crpP*-like	*catB7*		*aph(3′)-IIb*			
180	ST 856			*fosA*					*catB7*		*aph(3′)-IIb*			
183	ST 244			*fosA*					*catB7*		*aph(3′)-IIb*-like			
186	ST 3588			*fosA*-like					*catB7*-like		*aph(3′)-IIb*-like			
190	ST 871			*fosA*					*catB7*-like		*aph(3′)-IIb*			
195	ST 988			*fosA*				*crpP*-like	*catB7*-like		*aph(3′)-IIb*-like			
196	ST 2475			*fosA*				*crpP*-like	*catB7*		*aph(3′)-IIb*			
198	ST 2476			*fosA*				*crpP*-like	*catB7*		*aph(3′)-IIb*			
204	ST 639			*fosA*				*crpP*	*catB7*		*aph(3′)-IIb*-like			
208	ST 132			*fosA*				*crpP*-like	*catB7*		*aph(3′)-IIb*			
218	ST 856			*fosA*					*catB7*		*aph(3′)-IIb*			
229	ST 270			*fosA*				*crpP*-like	*catB7*		*aph(3′)-IIb*			
233	ST 3227			*fosA*					*catB7*		*aph(3′)-IIb*			
236	ST 266			*fosA*					*catB7*		*aph(3′)-IIb*			
238	ST 3589			*fosA*-like				*crpP*-like	*catB7*-like		*aph(3′)-IIb*-like			
242	ST 3590			*fosA*-like										
243	ST 3590			*fosA*-like					*catB7*-like		*aph(3′)-IIb*-like			
272	ST 2033			*fosA*					*catB7*-like		*aph(3′)-IIb*-like			
274	ST 2033			*fosA*					*catB7*-like		*aph(3′)-IIb*			
278	ST 988			*fosA*				*crpP*-like	*catB7*-like					
282	ST 554			*fosA*				*crpP*-like	*catB7*		*aph(3′)-IIb*			
285	ST 554			*fosA*					*catB7*		*aph(3′)-IIb*			
289	ST 1485			*fosA*					*catB7*		*aph(3′)-IIb*			
290	ST 1485			*fosA*					*catB7*		*aph(3′)-IIb*			
296	ST 235	*bla*_TEM-1B_, *bla*_SCO-1_	*sul1*	*fosA*			*tet(G)*		*catB7*-like		*aph(3′)-IIb*-like, *aac(3)-IIa*			
298	ST 3227			*fosA*					*catB7*		*aph(3′)-IIb*			
301	ST 3593			*fosA*-like					*catB7*-like		*aph(3′)-IIb*-like			
302	ST 1755			*fosA*					*catB7*		*aph(3′)-IIb*			
309	ST 3592			*fosA like*				*crpP*-like	*catB7*-like		*aph(3′)-IIb*-like			
310	ST 532		*sul1*	*fosA*					*catB7*-like		*aph(3″)-Ib, aph(6)-Id, aph(3′)-IIb*			
312	ST 381			*fosA*					*catB7*		*aph(3′)-IIb*			

Acquired resistance genes for macrolides, rifampicin, resistance-associated point mutations, genes for efflux pumps or genes mediating tolerance against disinfectants were not detected.

**Table A2:** Analysis of antimicrobial resistance determinants, ordered by strain and MLST type, of the assessed *K. pneumoniae* isolates. ST = Sequence type.

Sample ID	ST-Type				Acquired Resistance Determinants Against									
		Beta Lacatams	Sulfonamids	Fosfomycin	Trimethoprim	Macrolides	Tetracyclines	Fluoroquinolones	Chloramphenicol	Rifampicin	Aminoglycosides	Efflux Pumps	Amino Acid Exchanges Due to Point Mutations	Disinfectant Resistance Genes *
044	ST 327			*fosA*				*oqxB, oqxA*					*ompK37* p.I70M, *ompK37* p.I128M,*ompK37* p.I128M, *ompK36* p.L59V, *ompK36* p.L191S, *ompK36* p.F207W, *ompK36* p.A217S, *ompK36* p.N218H, *ompK36* p.D224E, *ompK36* p.L228V, *ompK36* p.E232R, *ompK36* p.T254S, *acrR* p.P161R, *acrR* p.G164A, *acrR* p.F172S, *acrR* p.R173G, *acrR* p.L195V, *acrR* p.F197I, *acrR* p.K201M	*oqxB, oqxA*
060	ST 5379	*bla*_TEM-1C_	*sul1, sul2*	*fosA*	*dfrA12*	*mph(A)*		*oqxA, oqxB, qnrS1*	*catA2*-like		*aph(6)-Id, aph(3″)-Ib, aph(3′)-Ia, aadA2, aac(3)-IIa*		*acrR* p.P161R, *acrR* p.G164A, *acrR* p.F172S, *acrR* p.R173G, *acrR* p.L195V, *acrR* p.F197I, *acrR* p.K201M, *ompK36* p.N49S, *ompK36* p.L59V, *ompK36* p.T184P, *ompK37* p.I70M, *ompK37* p.I128M	*oqxA, qacE, oqxB*
073	ST 39	*bla*_TEM-1B_ , *bla*_CTX-M-15_	*sul1,*	*fosA*	*dfrA27*	*erm(B), mph(A)*	*tet(D)*	*oqxB, oqxA, aac(6′)-Ib-cr, qnrB2, aac(6′)-Ib-cr*	*catA2*-like	*ARR-3*	*aac(6′)-Ib-cr, aadA16, aac(3)-IIa, aac(6′)-Ib-cr, aph(3″)-Ib, aph(6)-Id*		*acrR* p.P161R, *acrR* p.G164A, *acrR* p.F172S, *acrR* p.R173G, *acrR* p.L195V, *acrR* p.F197I, *acrR* p.K201M, *ompK37* p.I70M, *ompK37* p.I128M, *ompK37* p.N230G, *ompK36* p.N49S, *ompK36* p.L59V, *ompK36* p.L191S, *ompK36* p.F207W, *ompK36* p.A217S, *ompK36* p.N218H, *ompK36* p.D224E, *ompK36* p.L228V, *ompK36* p.E232R, *ompK36* p.T254S	*oqxB, oqxA, qacE*
100	ST 152	*bla*_CTX-M-15_ , *bla*_OXA-1_ , *bla*_TEM-1B_	*sul2, sul1*	*fosA*	*dfrA1, dfrA27*	*mph(A)*	*tet(D)*	*aac(6′)-Ib-cr, oqxB, qnrB6, oqxA, aac(6′)-Ib-cr*	*catB3, catA1, catB3*	*ARR-3*	*aac(3)-IIa, aph(6)-Id, aph(3″)-Ib, aadA1, aadA16, aph(3′)-Ia, aac(6′)-Ib-cr, aac(6′)-Ib-cr*		*ompK36* p.N49S, *ompK36* p.L59V, *ompK36* p.G189T, *ompK36* p.F198Y, *ompK36* p.F207Y, *ompK36* p.A217S, *ompK36* p.T222L, *ompK36* p.D223G, *ompK36* p.E232R, *ompK36* p.N304E, *acrR* p.P161R, *acrR* p.G164A, *acrR* p.F172S, *acrR* p.R173G, *acrR* p.L195V, *acrR* p.F197I, *acrR* p.K201M, *ompK37* p.I70M, *ompK37* p.I128M, *ompK37* p.N230G	*oqxB, oqxA*
102	ST 514			*fosA*			*tet(C)*	*oqxB, oqxA*	*catA1*				*ompK36* p.N49S, *ompK36* p.L59V, *ompK36* p.L191S, *ompK36* p.F207W, *ompK36* p.A217S, *ompK36* p.N218H, *ompK36* p.D224E, *ompK36* p.L228V, *ompK36* p.E232R, *ompK36* p.T254S, *ompK37* p.I70M, *ompK37* p.I128M, *ompK37* p.N230G, *acrR* p.P161R, *acrR* p.G164A, *acrR* p.F172S, acrR p.R173G, *acrR* p.L195V, *acrR* p.F197I, *acrR* p.K201M	*oqxB, oqxA*
124	ST 399			*fosA*				*oqxA, oqxB*	*catA1*				*ompK36* p.N49S, *ompK36* p.L59V, *ompK36* p.G189T, *ompK36* p.F198Y, *ompK36* p.F207Y, *ompK36* p.A217S, *ompK36* p.T222L, *ompK36* p.D223G, *ompK36* p.E232R, *acrR* p.P161R, *acrR* p.G164A, *acrR* p.F172S, *acrR* p.R173G, *acrR* p.F197I, *acrR* p.K201M, *ompK37* p.I70M, *ompK37* p.I128M	*oqxA, oqxB*
146	ST 4		*sul2*	*fosA*			*tet(D)*	*oqxA, oqxB*	*catA2*-like					*oqxA, oqxB*
177	ST 17		*sul1, sul2*	*fosA*	*dfrA15*		*tet(A)*	*oqxA, oqxB*-like	*catA1*		*aadA1, aph(3″)-Ib, aph(6)-Id*		*ompK37* p.I70M, *ompK37* p.I128M, *acrR* p.P161R, *acrR* p.G164A, *acrR* p.F172S, *acrR* p.R173G, *acrR* p.L195V, *acrR* p.F197I, *acrR* p.K201M, *ompK36* p.N49S, *ompK36* p.L59V,*ompK36* p.L191S, *ompK36* p.F207W, *ompK36* p.A217S, *ompK36* p.N218H, *ompK36* p.D224E, *ompK36* p.L228V, *ompK36* p.E232R*ompK36* p.T254S	*qacE, oqxB*-like, *oqxA*
181	ST 5380			*fosA*				*oqxA, oqxB*					*ompK36* p.N49S, *ompK36* p.L59V, *ompK36* p.L191S, *ompK36* p.F207W, *ompK36* p.A217S, *ompK36* p.N218H, *ompK36* p.D224E, *ompK36* p.L228V, *ompK36* p.E232R, *ompK36* p.T254S,*ompK37* p.I70M, *ompK37* p.I128M, *acrR* p.P161R, *acrR* p.G164A, *acrR* p.F172S, *acrR* p.R173G, *acrR* p.L195V, *acrR* p.F197I, *acrR* p.K201M	*oqxA, oqxB*
184	ST 5381			*fosA*				*oqxA*-like, *oqxB*-like					*ompK37* p.I70M, *ompK37* p.I128M, *ompK36* p.N49S, *ompK36* p.L59V, *ompK36* p.L191Q, *ompK36* p.F198Y,*ompK36* p.A217S, *ompK36* p.N218H, *ompK36* p.Q227N, *ompK36* p.L229V, *ompK36* p.N304E, *acrR* p.P161R, *acrR* p.G164A, *acrR* p.F172S, *acrR* p.R173G, *acrR* p.L195V, *acrR* p.F197I, *acrR* p.K201M	*oqxA*-like, *oqxB*-like
199	ST 17	*bla*_CTX-M-15_ , *bla*_TEM-1B_	*sul2, sul1*	*fosA*-like	*dfrA16*			*oqxA, oqxB*			*aadA2b, aac(3)-IIa*		*acrR* p.P161R, *acrR* p.G164A, *acrR* p.F172S, *acrR* p.R173G, acrR p.L195V, *acrR* p.F197I, *acrR* p.K201M, *ompK37* p.I70M, *ompK37* p.I128M, *ompK36* p.N49S, *ompK36* p.L59V, *ompK36* p.T86V, *ompK36* p.S89T, *ompK36* p.D91K, *ompK36* p.A93S, *ompK36* p.L191Q, *ompK36* p.F207W, *ompK36* p.A217S, *ompK36* p.N218H, *ompK36* p.Q227N, *ompK36* p.L229V, *ompK36* p.E232R,*ompK36* p.H235D, *ompK36* p.T254S	*oqxA, oqxB, qacE*
214	ST 6		*sul1*	*fosA*-like	*dfrA14*			*oqxB*-like, *oqxA*	*catA1*		*aph(3′)-Ia*		*ompK37* p.I70M, *ompK37* p.I128M, *ompK36* p.N49S, *ompK36* p.L59V, *ompK36* p.G189T, *ompK36* p.F198Y, *ompK36* p.F207Y, *ompK36* p.A217S, *ompK36* p.T222L, *ompK36* p.D223G, *ompK36* p.E232R, *acrR* p.P161R, *acrR* p.G164A, *acrR* p.F172S, *acrR* p.R173G, *acrR* p.L195V, *acrR* p.F197I, *acrR* p.K201M	*oqxB*- like, *oqxA*
217	ST 3154	*bla*_SCO-1_ , *bla*_TEM-1B_	*sul1, sul2*	*fosA*	*dfrA12, dfrA14*		*tet(A)*	*oqxA, oqxB*-like	*catA2*-like		*aph(6)-Id, aph(3″)-Ib, aac(3)-IIa, aadA2*		*acrR* p.P161R, *acrR* p.G164A, *acrR* p.F172S, *acrR* p.R173G, *acrR* p.L195V, *acrR* p.F197I, *acrR* p.K201M, *ompK37* p.I70M, *ompK37* p.I128M, *ompK36* p.N49S, *ompK36* p.L59V, *ompK36* p.G189T, *ompK36* p.F198Y, *ompK36* p.F207Y, *ompK36* p.A217S, *ompK36* p.T222L, *ompK36* p.D223G, *ompK36* p.E232R, *ompK36* p.N304E	*oqxA, qacE, oqxB*-like
220	ST 5382			*fosA*-like				*oqxB*-like, *oqxA*-like	*catA1*				*ompK37* p.I70M, *ompK37* p.I128M, *ompK36* p.N49S, *ompK36* p.L59V, *ompK36* p.L191Q, *ompK36* p.A217S, *ompK36* p.N218H, *ompK36* p.Q227N, *ompK36* p.L229V, *ompK36* p.N304E, *acrR* p.P161R, *acrR* p.F172S, *acrR* p.R173G, *acrR* p.L195V, *acrR* p.F197I, *acrR* p.K201M	*oqxB*-like, *oqxA*-like
234	ST 109			*fosA*				*oqxA, oqxB*-like					*ompK36* p.N49S, *ompK36* p.L59V, *ompK36* p.L191S, *ompK36* p.F207W, *ompK36* p.A217S, *ompK36* p.N218H, *ompK36* p.D224E, *ompK36* p.L228V, *ompK36* p.E232R, *ompK36* p.T254S, *acrR* p.P161R, *acrR* p.G164A, *acrR* p.F172S,*acrR* p.R173G, *acrR* p.L195V, *acrR* p.F197I, *acrR* p.K201M, *ompK37* p.I70M, *ompK37* p.I128M	*oqxA, oqxB*-like
240	ST 5383			*fosA*-like			*tet(D)*	*oqxA, oqxB*-like					*ompK36* p.N49S, *ompK36* p.L59V, *ompK36* p.L191S, *ompK36* p.F207W, *ompK36* p.A217S, *ompK36* p.N218H, *ompK36* p.D224E, *ompK36* p.L228V, *ompK36* p.E232R, *ompK37* p.I70M, *ompK37* p.I128M, *acrR* p.P161R, *acrR* p.G164A, *acrR* p.F172S, *acrR* p.R173G, *acrR* p.L195V, *acrR* p.F197I,*acrR* p.K201M	*oqxA, oqxB*-like
248	ST 5384			*fosA*-like			*tet(A)*	*oqxB*-like, *oqxA*-like	*catA1*				*ompK36* p.N49S, *ompK36* p.L59V, *ompK36* p.L191S, *ompK36* p.F198Y, *ompK36* p.F207W, *ompK36* p.A217S, *ompK36* p.N218H, *ompK36* p.D224E, *ompK36* p.L228V, *ompK36* p.E232R, *ompK37* p.I70M, *ompK37* p.I128M, *acrR* p.P161R, *acrR* p.G164A, *acrR* p.F172S, *acrR* p.R173G, *acrR* p.L195V, *acrR* p.F197I, *acrR* p.K201M	*oqxB*-like, *oqxA*-like
252	ST 607	*bla*_TEM-1B_	*sul2, sul1*	*fosA*-like	*dfrA7*		*tet(A)*	*oqxB*-like, *oqxA*	*catA1*		*aph(3″)-Ib, aph(6)-Id*		*ompK37* p.I70M, *ompK37* p.I128M, *ompK37* p.N230G, *ompK36* p.N49S, *ompK36* p.L59V, *ompK36* p.L191S, *ompK36* p.F207W, *ompK36* p.A217S, *ompK36* p.N218H, ompK36 p.D224E, *ompK36* p.L228V, *ompK36* p.E232R, *ompK36* p.T254S, *acrR* p.P161R,*acrR* p.G164A, *acrR* p.F172S, *acrR* p.R173G, *acrR* p.L195V, *acrR* p.F197I, *acrR* p.K201M	*oqxB*-like, *oqxA, qacE*
267	ST 36	*bla*_CTX-M-15_ , *bla*_TEM-1B_	*sul2, sul1*	*fosA*	*dfrA27*		*tet(D)*	*aac(6′)-Ib-cr, oqxA, oqxB*	*catA2*-like	*ARR-3*	*aph(6)-Id, aph(3″)-Ib, aac(6′)-Ib-cr, aadA16, aac(3)-IIa, aph(6)-Id*		*ompK36* p.N49S, *ompK36* p.L59V, *ompK36* p.T184P, *ompK37* p.I70M, *ompK37* p.I128M, *ompK37* p.N230G, *acrR* p.P161R, *acrR* p.G164A, *acrR* p.F172S, *acrR* p.R173G, *acrR* p.L195V, *acrR* p.F197I, *acrR* p.K201M	*oqxA, qacE, oqxB*
277	ST 530	*bla*_TEM-35_	*sul2*	*fosA*-like	*dfrA14*		*tet(D)*	*oqxA, oqxB*-like			*aph(3″)-Ib, aph(6)-Id*		*acrR* p.P161R, *acrR* p.G164A, *acrR* p.F172S, *acrR* p.R173G, *acrR* p.L195V, *acrR* p.F197I, *acrR* p.K201M, *ompK36* p.N49S, *ompK36* p.L59V, *ompK36* p.L191S, *ompK36* p.F207W, *ompK36* p.A217S, *ompK36* p.N218H, *ompK36* p.D224E, *ompK36* p.L228V, *ompK36* p.E232R,*ompK36* p.T254S, *ompK37* p.I70M, *ompK37* p.I128M	*oqxA, oqxB*-like
279	ST 5385			*fosA*				*oqxA, oqxB*					*acrR* p.P161R, *acrR* p.G164A, *acrR* p.F172S, *acrR* p.R173G, *acrR* p.L195V, *acrR* p.F197I, *acrR* p.K201M, *ompK36* p.N49S, *ompK36* p.L59V, *ompK36* p.T184P, *ompK37* p.I70M, *ompK37* p.I128M	*oqxA, oqxB*

* *qacE* = quaternary ammonium compounds resistance and *oqxB* and *oqxA* = efflux pumps mediating resistance against disinfectants.

**Table A3:** Phenotypic resistance the *P. aeruginosa* strains. Data are missing for strains 198, 218 and 312, due to loss during subcultivation. MIC = minimum inhibitory concentration. N.a. = value missing due to loss of strain or failed reaction.

Sample ID	Piperacillin		Piperacillin/Tazobactam		Ceftrazidime		Cefepime		Imipenem		Meropenem		Gentamicin	
	MIC	Interpretation	MIC	Interpretation	MIC	Interpretation	MIC	Interpretation	MIC	Interpretation	MIC	Interpretation	MIC	Interpretation
17	≤4	S	≤4	S	≤1	S	≤1	S	≤0.25	S	≤0.25	S	≤1	S
22	≤4	S	≤4	S	≤1	S	≤1	S	≤0.25	S	≤0.25	S	≤1	S
32	≤4	S	8	S	2	S	2	S	1	S	≤0.25	S	≤1	S
69	≤4	S	≤4	S	4	S	2	S	1	S	≤0.25	S	≤1	S
81	≤4	S	8	S	2	S	≤1	S	1	S	1	S	≤1	S
82	16	S	8	S	4	S	2	S	2	S	≤0.25	S	≤1	S
88	≥128	R	≥128	R	≥64	R	32	R	≥16	R	4	I	≤1	S
99	8	S	8	S	4	S	2	S	2	S	1	S	≤1	S
106	≤4	S	8	S	2	S	2	S	2	S	1	S	≤1	S
114	≤4	S	8	S	2	S	≤1	S	2	S	1	S	≤1	S
137	16	S	8	S	4	S	2	S	2	S	2	S	≤1	S
144	16	S	8	S	4	S	2	S	2	S	1	S	≤1	S
147	8	S	≤4	S	4	S	8	S	2	S	0.5	S	4	S
149	8	S	8	S	4	S	2	S	≤0.25	S	≤0.25	S	≤1	S
153	≤4	S	8	S	2	S	≤1	S	1	S	≤0.25	S	≤1	S
154	64	R	≤4	S	≤1	S	≤1	S	2	S	0.5	S	≤1	S
157	16	S	8	S	4	S	4	S	2	S	≤0.25	S	2	S
160	≥128	R	32	R	16	R	32	R	8	I	8	I	8	R
162	64	R	32	R	8	S	8	S	2	S	1	S	2	S
180	16	S	8	S	4	S	2	S	2	S	≤0.25	S	≤1	S
183	8	S	8	S	4	S	2	S	2	S	0.5	S	≤1	S
186	16	n.a.	n.a.	S	4	S	2	S	2	S	≤0.25	S	≤1	S
190	16	S	8	S	4	S	2	S	2	S	0.5	S	≤1	S
195	8	S	8	S	4	S	≤1	S	2	S	≤0.25	S	≤1	S
196	≤4	S	≤4	S	2	S	≤1	S	2	S	0.5	S	≤1	S
198	n.a.	n.a.	n.a.	n.a.	n.a.	n.a.	n.a.	n.a.	n.a.	n.a.	n.a.	n.a.	n.a.	n.a.
204	8	S	8	S	2	S	≤1	S	2	S	≤0.25	S	≤1	S
208	8	S	8	S	4	S	2	S	2	S	≤0.25	S	≤1	S
218	n.a.	n.a.	n.a.	n.a.	n.a.	n.a.	n.a.	n.a.	n.a.	n.a.	n.a.	n.a.	n.a.	n.a.
229	8	S	8	S	4	S	2	S	1	S	≤0.25	S	≤1	S
233	≥128	R	≥128	R	32	R	8	S	8	I	4	I	≤1	S
236	16	S	16	S	4	S	2	S	2	S	0.5	S	2	S
238	8	S	16	S	4	S	2	S	2	S	≤0.25	S	≤1	S
242	8	S	8	S	4	S	2	S	1	S	≤0.25	S	≤1	S
243	16	S	8	S	4	S	2	S	1	S	≤0.25	S	≤1	S
272	64	R	64	R	8	S	4	S	2	S	1	S	≤1	S
274	16	S	8	S	4	S	2	S	2	S	0.5	S	≤1	S
278	≤4	S	≤4	S	2	S	≤1	S	2	S	≤0.25	S	≤1	S
282	≤4	S	8	S	≤1	S	≤1	S	2	S	1	S	≤1	S
285	≤4	S	≤4	S	≤1	S	≤1	S	2	S	1	S	≤1	S
289	≤4	S	8	S	≤1	S	≤1	S	2	S	1	S	≤1	S
290	8	S	8	S	≤1	S	≤1	S	2	S	1	S	≤1	S
296	≥128	R	64	R	4	S	8	S	1	S	1	S	≥16	R
298	≥128	R	≥128	R	≥64	R	8	S	8	I	4	I	≤1	S
301	16	S	8	S	4	S	2	S	2	S	0.5	S	≤1	S
302	8	S	8	S	4	S	≤1	S	2	S	0.5	S	≤1	S
309	16	S	8	S	4	S	4	S	2	S	1	S	≤1	S
310	32	R	16	S	4	S	4	S	2	S	1	S	≤1	S
312	n.a.	n.a.	n.a.	n.a.	n.a.	n.a.	n.a.	n.a.	n.a.	n.a.	n.a.	n.a.	n.a.	n.a.

**Table A4:** Phenotypic resistance of *P. aeruginosa* strains. Data are missing for strains 198, 218 and 312 due to loss during subcultivation. MIC = minimum inhibitory concentration. N.a. = value missing due to loss of strain or failed reaction.

Sample ID	Ciprofloxacin		Moxifloxacin		Aztreonam		Amikacin		Tobramycin		Fosfomycin		Colistin	
	MIC	Interpretation	MIC	Interpretation	MIC	Interpretation	MIC	Interpretation	MIC	Interpretation	MIC	Interpretation	MIC	Interpretation
17	≤0.25	S	1	R	4	I	≤2	S	≤1	S	128	R	≤0.5	S
22	≤0.25	S	1	R	2	I	≤2	S	≤1	S	128	R	1	S
32	≤0.25	S	2	R	4	I	≤2	S	≤1	S	128	R	≤0.5	S
69	≤0.25	S	0.5	R	4	I	≤2	S	≤1	S	128	R	≤0.5	S
81	≤0.25	S	0.5	R	4	I	≤2	S	≤1	S	≥256	R	≤0.5	S
82	≤0.25	S	1	R	16	I	≤2	S	≤1	S	≤16	R	≤0.5	S
88	2	R	≥8	R	32	R	≤2	S	≤1	S	128	R	≤0.5	S
99	≤0.25	S	2	R	16	I	≤2	S	≤1	S	128	R	≤0.5	S
106	2	R	1	R	8	I	≤2	S	≤1	S	≥256	R	≤0.5	S
114	≤0.25	S	0.5	R	4	I	≤2	S	≤1	S	128	R	≤0.5	S
137	≤0.25	S	1	R	16	I	≤2	S	≤1	S	≥256	R	≤0.5	S
144	≤0.25	S	1	R	16	I	≤2	S	≤1	S	≥256	R	≤0.5	S
147	≤0.25	S	2	R	4	I	8	S	≤1	S	≥256	R	≤0.5	S
149	≤0.25	S	1	R	16	I	≤2	S	≤1	S	≥256	R	≤0.5	S
153	≤0.25	S	2	R	4	I	≤2	S	≤1	S	≤16	R	≤0.5	S
154	≤0.25	S	0.5	R	4	I	≤2	S	≤1	S	≥256	R	≤0.5	S
157	≤0.25	S	1	R	16	I	≤2	S	≤1	S	≥256	R	≤0.5	S
160	1	R	≥8	R	≥64	R	16	I	≤1	S	64	R	≤0.5	S
162	0.5	S	2	R	32	R	4	S	≤1	S	128	R	≤0.5	S
180	≤0.25	S	1	R	16	I	≤2	S	≤1	S	≥256	R	≤0.5	S
183	≤0.25	S	0.5	R	4	I	≤2	S	≤1	S	≥256	R	≤0.5	S
186	≤0.25	S	1	R	16	I	≤2	S	≤1	S	32	R	≤0.5	S
190	≤0.25	S	1	R	16	I	4	S	≤1	S	32	R	≤0.5	S
195	≤0.25	S	0.5	R	4	I	≤2	S	≤1	S	64	R	≤0.5	S
196	≤0.25	S	0.5	R	4	I	≤2	S	≤1	S	64	R	≤0.5	S
198	n.a.	n.a.	n.a.	n.a.	n.a.	n.a.	n.a.	n.a.	n.a.	n.a.	n.a.	n.a.	n.a.	n.a.
204	≤0.25	S	1	R	2	I	≤2	S	≤1	S	32	R	2	S
208	≤0.25	S	1	R	8	I	≤2	S	≤1	S	128	R	≤0.5	S
218	n.a.	n.a.	n.a.	n.a.	n.a.	n.a.	n.a.	n.a.	n.a.	n.a.	n.a.	n.a.	n.a.	n.a.
229	≤0.25	S	1	R	4	I	≤2	S	≤1	S	128	R	≤0.5	S
233	≤0.25	S	2	R	16	I	≤2	S	≤1	S	≤16	R	≤0.5	S
236	≤0.25	S	1	R	16	I	8	S	≤1	S	64	R	≤0.5	S
238	≤0.25	S	2	R	8	I	≤2	S	≤1	S	≤16	R	≤0.5	S
242	≤0.25	S	1	R	8	I	≤2	S	≤1	S	128	R	≤0.5	S
243	≤0.25	S	2	R	16	I	≤2	S	≤1	S	64	R	≤0.5	S
272	≤0.25	S	2	R	32	R	≤2	S	≤1	S	≥256	R	≤0.5	S
274	≤0.25	S	2	R	16	I	≤2	S	≤1	S	≥256	R	≤0.5	S
278	≤0.25	S	0.5	R	4	I	≤2	S	≤1	S	128	R	≤0.5	S
282	≤0.25	S	1	R	2	I	≤2	S	≤1	S	128	R	≤0.5	S
285	≤0.25	S	1	R	2	I	≤2	S	≤1	S	128	R	≤0.5	S
289	≤0.25	S	0.5	R	4	I	≤2	S	≤1	S	128	R	≤0.5	S
290	≤0.25	S	0.5	R	4	I	≤2	S	≤1	S	128	R	≤0.5	S
296	≥4	R	≥8	R	32	R	≤2	S	≥16	R	64	R	≤0.5	S
298	≤0.25	S	2	R	16	I	≤2	S	≤1	S	≤16	R	≤0.5	S
301	≤0.25	S	2	R	16	I	≤2	S	≤1	S	≤16	R	≤0.5	S
302	≤0.25	S	1	R	4	I	≤2	S	≤1	S	128	R	≤0.5	S
309	≤0.25	S	1	R	4	I	≤2	S	≤1	S	≤16	R	≤0.5	S
310	≤0.25	S	2	R	16	I	≤2	S	≤1	S	128	R	≤0.5	S
312	n.a.	n.a.	n.a.	n.a.	n.a.	n.a.	n.a.	n.a.	n.a.	n.a.	n.a.	n.a.	n.a.	n.a.

**Table A5:** Phenotypic resistance of the *Klebsiella* strains. MIC = minimum inhibitory concentration. ESBL = signal in phenotypic testing for extended-spectrum beta-lactamases.

Sample ID	ESBL	Ampicillin		Ampicillin/Sulbactam		Piperacillin/Tazobactam		Cefuroxime		Cefuroxime Axetil		Cefpodoxime		Cefotaxime		Ceftrazidime	
		MIC	Interpretation	MIC	Interpretation	MIC	Interpretation	MIC	Interpretation	MIC	Interpretation	MIC	Interpretation	MIC	Interpretation	MIC	Interpretation
44	negative	≥32	R	16	R	≤4	S	4	I	4	I	≤0.25	S	≤1	S	≤1	S
60	negative	≥32	R	16	R	≤4	S	≤1	I	≤1	S	≤0.25	S	≤1	S	≤1	S
73	positive	≥32	R	≥32	R	≥128	R	≥64	R	≥64	R	≥8	R	≥64	R	8	R
100	positive	≥32	R	≥32	R	≥128	R	≥64	R	≥64	R	≥8	R	≥64	R	16	R
102	negative	≥32	R	≤2	I	8	S	8	I	8	I	≤0.25	S	≤1	S	≤1	S
124	negative	≥32	R	≤2	I	≤4	S	2	I	2	I	≤0.25	S	≤1	S	≤1	S
146	negative	≥32	R	≤2	I	8	S	2	I	2	I	≤0.25	S	≤1	S	≤1	S
177	positive	≥32	R	≥32	R	≥128	R	2	I	2	S	≤0.25	S	≤1	S	≤1	I
181	negative	≥32	R	≤2	I	≤4	S	2	I	2	I	≤0.25	S	≤1	S	≤1	S
184	negative	16	R	≤2	I	≤4	S	2	I	2	S	≤0.25	S	≤1	S	≤1	S
199	positive	≥32	R	≥32	R	8	R	≥64	R	≥64	R	≥8	R	≥64	R	16	R
214	negative	≥32	R	≤2	I	≤4	S	≤1	I	≤1	S	≤0.25	S	≤1	S	≤1	S
217	negative	≥32	R	≥32	R	≥128	R	4	I	4	I	≤0.25	S	≤1	S	≤1	S
220	negative	≥32	R	≤2	I	≤4	S	4	I	4	I	≤0.25	S	≤1	S	≤1	S
234	negative	≥32	R	≤2	I	≤4	S	2	I	2	I	≤0.25	S	≤1	S	≤1	S
240	negative	≥32	R	≤2	I	≤4	S	≤1	I	≤1	S	≤0.25	S	≤1	S	≤1	S
248	negative	≥32	R	≤2	I	≤4	S	2	I	2	I	≤0.25	S	≤1	S	≤1	S
252	negative	≥32	R	16	R	≤4	S	2	I	2	I	≤0.25	S	≤1	S	≤1	S
267	positive	≥32	R	≥32	R	32	R	≥64	R	≥64	R	≥8	R	≥64	R	16	R
277	negative	≥32	R	≥32	R	64	R	2	I	2	I	≤0.25	S	≤1	S	≤1	S
279	negative	≥32	R	≤2	I	≤4	S	2	I	2	I	≤0.25	S	≤1	S	≤1	S

**Table A6:** Phenotypic resistance of the *Klebsiella* strains. MIC = minimum inhibitory concentration. ESBL = signal in phenotypic testing for extended-spectrum beta-lactamases.

Sample ID	ESBL	Ertapenem		Imipenem		Meropenem		Gentamicin		Ciprofloxacin		Moxifloxacin		Tigecycline		Trimethoprim/Sulfamethoxazole	
		MIC	Interpretation	MIC	Interpretation	MIC	Interpretation	MIC	Interpretation	MIC	Interpretation	MIC	Interpretation	MIC	Interpretation	MIC	Interpretation
44	negative	≤0.5	S	≤0.25	S	≤0.25	S	≤1	S	≤0.25	S	≤0.25	S	≤0.5	S	≤20	S
60	negative	≤0.5	S	≤0.25	S	≤0.25	S	≥16	R	1	R	2	R	≤0.5	S	≥320	R
73	positive	≤0.5	S	≤0.25	S	≤0.25	S	≥16	R	1	R	2	R	≤0.5	S	≥320	R
100	positive	≤0.5	S	≤0.25	S	≤0.25	S	≥16	R	≥4	R	≥8	R	≤0.5	S	≥320	R
102	negative	≤0.5	S	≤0.25	S	≤0.25	S	≤1	S	≤0.25	S	0.5	R	4	R	≤20	S
124	negative	≤0.5	S	≤0.25	S	≤0.25	S	≤1	S	≤0.25	S	≤0.25	S	≤0.5	S	≤20	S
146	negative	≤0.5	S	≤0.25	S	≤0.25	S	≤1	S	≤0.25	S	≤0.25	S	1	S	≤20	S
177	negative	≤0.5	S	≤0.25	S	≤0.25	S	≤1	S	≤0.25	S	≤0.25	S	2	I	≥320	R
181	negative	≤0.5	S	≤0.25	S	≤0.25	S	≤1	S	≤0.25	S	≤0.25	S	≤0.5	S	≤20	S
184	negative	≤0.5	S	≤0.25	S	≤0.25	S	≤1	S	≤0.25	S	≤0.25	S	≤0.5	S	≤20	S
199	positive	≤0.5	S	≤0.25	S	≤0.25	S	≥16	R	≤0.25	S	≤0.25	S	≤0.5	S	≥320	R
214	negative	≤0.5	S	≤0.25	S	≤0.25	S	≤1	S	≤0.25	S	≤0.25	S	≤0.5	S	≥320	R
217	negative	≤0.5	S	≤0.25	S	≤0.25	S	≥16	R	≤0.25	S	≤0.25	S	2	I	≥320	R
220	negative	≤0.5	S	≤0.25	S	≤0.25	S	≤1	S	≤0.25	S	0.5	R	1	S	≤20	S
234	negative	≤0.5	S	≤0.25	S	≤0.25	S	≤1	S	≤0.25	S	0.5	R	1	S	≤20	S
240	negative	≤0.5	S	≤0.25	S	≤0.25	S	≤1	S	≤0.25	S	≤0.25	S	≤0.5	S	≤20	S
248	negative	≤0.5	S	≤0.25	S	≤0.25	S	≤1	S	≤0.25	S	≤0.25	S	2	I	≤20	S
252	negative	≤0.5	S	≤0.25	S	≤0.25	S	≤1	S	≤0.25	S	≤0.25	S	1	S	≥320	R
267	positive	≤0.5	S	≤0.25	S	≤0.25	S	≥16	R	≤0.25	S	≤0.25	S	≤0.5	S	≥320	R
277	negative	≤0.5	S	≤0.25	S	≤0.25	S	≤1	S	≤0.25	S	≤0.25	S	1	S	≥320	R
279	negative	≤0.5	S	≤0.25	S	≤0.25	S	≤1	S	≤0.25	S	≤0.25	S	≤0.5	S	≤20	S

**Table A7:** Phenotypic resistance of *Escherichia coli* strains. MIC = minimum inhibitory concentration. ESBL = signal in phenotypic testing for extended-spectrum beta-lactamases.

Sample ID	ESBL	Ampicillin		Ampicillin/Sulbactam		Piperacillin/Tazobactam		Cefuroxime		Cefuroxime Axetil		Cefpodoxime		Cefotaxime		Ceftrazidime	
		MIC	Interpretation	MIC	Interpretation	MIC	Interpretation	MIC	Interpretation	MIC	Interpretation	MIC	Interpretation	MIC	Interpretation	MIC	Interpretation
41	positive	≥32	R	≥32	R	64	R	≥64	R	≥64	R	≥8	R	≥64	R	16	R
49	negative	≥32	R	16	R	≤4	S	4	I	4	S	≤0.25	S	≤1	S	≤1	S
68	negative	≥32	R	16	R	≤4	S	≤1	I	≤1	S	≤0.25	S	≤1	S	≤1	S
117	negative	≥32	R	≥32	R	64	R	4	I	4	S	≤0.25	S	≤1	S	≤1	S
152	negative	≥32	R	≥32	R	≥128	R	4	I	4	S	0.5	S	≤1	S	≤1	S
176	negative	≥32	R	≥32	R	≤4	I	2	I	2	S	≤0.25	S	≤1	S	≤1	S
221	negative	≥32	R	≥32	R	≤4	I	2	I	2	S	≤0.25	S	≤1	S	≤1	S
222	negative	≥32	R	≥32	R	≤4	I	4	I	4	S	≤0.25	S	≤1	S	≤1	S
225	positive	≥32	R	≥32	R	≤4	R	≥64	R	≥64	R	≥8	R	≥64	R	16	R
245	positive	≥32	R	≥32	R	16	I	16	R	16	R	1	S	2	I	≤1	S
270	positive	≥32	R	16	R	≤4	R	≥64	R	≥64	R	≥8	R	≥64	R	≥64	R
299	negative	≥32	R	≤2	I	≤4	S	4	I	4	S	≤0.25	S	≤1	S	≤1	S

**Table A8:** Phenotypic resistance of *Escherichia coli* strains. MIC = minimum inhibitory concentration. ESBL = signal in phenotypic testing for extended-spectrum beta-lactamases.

Sample ID	ESBL	Ertapenem		Imipenem		Meropenem		Gentamicin		Ciprofloxacin		Moxifloxacin		Tigecycline		Trimethoprim/Sulfamethoxazole	
		MIC	Interpretation	MIC	Interpretation	MIC	Interpretation	MIC	Interpretation	MIC	Interpretation	MIC	Interpretation	MIC	Interpretation	MIC	Interpretation
41	positive	≤0.5	S	≤0.25	S	≤0.25	S	≥16	R	≥4	R	≥8	R	≤0.5	S	≥320	R
49	negative	≤0.5	S	≤0.25	S	≤0.25	S	≤1	S	≤0.25	S	≤0.25	S	≤0.5	S	≥320	R
68	negative	≤0.5	S	≤0.25	S	≤0.25	S	≤1	S	≥4	R	≥8	R	≤0.5	S	≥320	R
117	negative	≤0.5	S	≤0.25	S	≤0.25	S	≤1	S	≤0.25	S	≤0.25	S	≤0.5	S	≥320	R
152	negative	≤0.5	S	≤0.25	S	≤0.25	S	2	S	1	R	2	R	≤0.5	S	≥320	R
176	negative	≤0.5	S	≤0.25	S	≤0.25	S	≤1	S	≤0.25	S	≤0.25	S	≤0.5	S	≥320	R
221	negative	≤0.5	S	≤0.25	S	≤0.25	S	≤1	S	≤0.25	S	≤0.25	S	≤0.5	S	≥320	R
222	negative	≤0.5	S	≤0.25	S	≤0.25	S	≤1	S	≤0.25	S	≤0.25	S	≤0.5	S	≥320	R
225	positive	≤0.5	S	≤0.25	S	≤0.25	S	≤1	S	0.5	I	1	R	≤0.5	S	≥320	R
245	positive	≤0.5	S	0.5	S	≤0.25	S	≥16	R	≥4	R	≥8	R	≤0.5	S	≥320	R
270	positive	≤0.5	S	≤0.25	S	≤0.25	S	≤1	S	≥4	R	≥8	R	≤0.5	S	≤20	S
299	negative	≤0.5	S	≤0.25	S	≤0.25	S	≤1	S	0.5	I	2	R	≤0.5	S	≥320	R

**Table A9:** Analysis of virulence determinants, ordered by strain and MLST type, of the assessed *P. aeruginosa* isolates. ST = Sequence type.

Sample ID	ST-Type	Pathogenicity Factor Groups								
		Adherence	Anti-Phagocytosis	Biosurfactant	Iron Uptake	Pigment	Protease	Toxin	Regulation	Secretion System
017	ST 381	*waaA, waaC, waaF, waaG, waaP, wzy, wzz, chpA, chpB, chpC, chpD, chpE, fimV, pilB, pilD, pilF, pilG, pilH, pilI, pilK, pilM, pilN, pilO, pilP, pilQ, pilR, pilS, pilT, pilU, pilC like, xcpA/pilD*	*alg44, alg8, algA, algB, algC, algD, algE, algF, algG, algP/algR3 algI, algJ, algK, algL, algQ, algR, algU, algW, algX, algZ, mucA, mucB, mucC*	*rhlA, rhlB*	*fptA, fpvA, pchA, pchB, pchC, pchD, pchE, pchF, pchG, pchH, pchI, pchR, pvdA, pvdD, pvdE*	*phzM, phzS*	*aprA, lasA*	*toxA, plcH*	*lasI, rhlI*	*xcpP, xcpQ, xcpR, xcpS, xcpT, xcpU, xcpV, xcpW, xcpX, xcpY, xcpZ*
022	ST 2483	*waaA, waaC, waaF, waaG, waaP, chpA, chpB, chpC, chpD, chpE, fimT, fimU, fimV, pilB, pilD, pilE, pilF, pilG, pilH, pilI, pilK, pilM, pilN, pilO, pilP, pilQ, pilR, pilS, pilT, pilU, pilV, pilW, pilX pilY1, pilY2, pilC, xcpA/pilD*	*alg44, alg8, algA, algB, algC, algD, algE, algF, algG, algI, algJ, algK, algL, algQ, algR, algU, algW, algX, algZ, mucA, mucB, mucC*	*rhlA, rhlB*	*fptA, pchA, pchB, pchC, pchD, pchE, pchF, pchG, pchH, pchI, pchR, pvdA*	*phzM, phzS*	*aprA, lasA*	*toxA, plcH*	*lasI, rhlI*	*xcpP, xcpQ, xcpR, xcpS, xcpT, xcpU, xcpV, xcpW, xcpX, xcpY, xcpZ*
032	ST 3587	*waaA, waaC, waaF, waaG, waaP, chpA, chpB, chpC, chpD, chpE, fimT, fimU, fimV, pilB, pilD, pilE, pilF, pilG, pilH, pilI, pilK, pilM, pilN, pilO, pilP, pilQ, pilR, pilS, pilT, pilU, pilV, pilW, pilX pilY1, pilY2, pilC like, xcpA/pilD*	*alg44, alg8, algA, algB, algC, algD, algE, algF, algG, algI, algJ, algK, algL, algQ, algR, algU, algW, algX, algZ, mucA, mucB, mucC*	*rhlA, rhlB*	*fptA, pchA, pchB, pchC, pchD, pchE, pchF, pchG, pchH, pchI, pchR, pvdA*	*phzM, phzS*	*aprA, lasA*	*toxA, plcH*	*lasI, rhlI*	*xcpP, xcpQ, xcpR, xcpS, xcpT, xcpU, xcpV, xcpW, xcpX, xcpY, xcpZ*
069	ST 360	*waaA, waaC, waaF, waaG, waaP, wzy, wzz, chpA, chpB, chpC, chpD, chpE, fimT, fimU, fimV, pilB, pilD, pilE, pilF, pilG, pilH, pilI, pilK, pilM, pilN, pilO, pilP, pilQ, pilR, pilS, pilT, pilU, pilV, pilW, pilX pilY1, pilY2, pilC like, xcpA/pilD*	*alg44, alg8, algA, algB, algC, algD, algE, algF, algG, algI, algJ, algK, algL, algQ, algR, algU, algW, algX, algZ, mucA, mucB, mucC*	*rhlA, rhlB*	*fptA, pchA, pchB, pchC, pchD, pchE, pchF, pchG, pchH, pchI, pchR, pvdA*	*phzM, phzS*	*aprA, lasA*	*toxA, plcH*	*lasI, rhlI*	*xcpP, xcpQ, xcpR, xcpS, xcpT, xcpU, xcpV, xcpW, xcpX, xcpY, xcpZ*
081	ST 244	*waaA, waaC, waaF, waaG, waaP, wzy, wzz, chpA, chpB, chpC, chpD, chpE, fimT, fimU, fimV, pilA like, pilB, pilD, pilE, pilF, pilG, pilH, pilI, pilK, pilM, pilN, pilO, pilP, pilQ, pilR, pilS, pilT, pilU, pilV, pilW, pilX pilY1, pilY2, pilC, xcpA/pilD*	*alg44, alg8, algA, algB, algC, algD, algE, algF, algG, algI, algJ, algK, algL, algQ, algR, algU, algW, algX, algZ, mucA, mucB, mucC*	*rhlA, rhlB*	*fptA, fpvA, pchA, pchB, pchC, pchD, pchE, pchF, pchG, pchH, pchI, pchR, pvdA, pvdE*	*phzM, phzS*	*aprA, lasA*	*toxA, plcH*	*lasI, rhlI*	*xcpP, xcpQ, xcpR, xcpS, xcpT, xcpU, xcpV, xcpW like, xcpX, xcpY, xcpZ*
082	ST 514	*waaA, waaC, waaF, waaG, waaP, chpA, chpB, chpC, chpD, chpE, fimV, pilB, pilD, pilF, pilG, pilH, pilI, pilK, pilM, pilN, pilO, pilP, pilQ, pilR, pilS, pilT, pilU, pilC, xcpA/pilD*	*alg44, alg8, algA, algB, algC, algD, algE, algF, algG, algI, algJ, algK, algL, algP/algR3, algQ, algR, algU, algW, algX, algZ, mucA, mucB, mucC*	*rhlA, rhlB*	*fptA, pchA, pchB, pchC, pchD, pchE, pchF, pchG, pchH, pchI, pchR, pvdA*	*phzM, phzS*	*aprA, lasA*	*toxA, plcH*	*lasI, rhlI*	*xcpP, xcpQ, xcpR, xcpS, xcpT, xcpU, xcpV, xcpW, xcpX, xcpY, xcpZ*
088	ST 1682	*waaA, waaC, waaF, waaG, waaP, wzy, wzz, chpA, chpB, chpC, chpD, chpE, fimT, fimU, fimV, pilB, pilD, pilE, pilF, pilG, pilH, pilI, pilK, pilM, pilN, pilO, pilP, pilQ, pilR, pilS, pilT, pilU, pilV, pilW, pilX pilY1, pilY2, xcpA/pilD*	*alg44, alg8, algA, algB, algC, algD, algE, algF, algG, algI, algJ, algK, algL, algQ, algR, algU, algW, algX, algZ, mucA, mucB, mucC*	*rhlA, rhlB*	*fptA, pchA, pchB, pchC, pchD, pchE, pchF, pchG, pchH, pchI, pchR, pvdA*	*phzM, phzS*	*aprA, lasA*	*toxA, plcH*	*lasI, rhlI*	*xcpP, xcpQ, xcpR, xcpS, xcpT, xcpU, xcpV, xcpW, xcpX, xcpY, xcpZ*
099	ST 244	*waaA, waaC, waaF, waaG, waaP, wzy, wzz, chpA, chpB, chpC, chpD, chpE, fimT, fimU, fimV, pilA, pilB, pilD, pilE, pilF, pilG, pilH, pilI, pilK, pilM, pilN, pilO, pilP, pilQ, pilR, pilS, pilT, pilU, pilV, pilW, pilX pilY1, pilY2, pilC, xcpA/pilD*	*alg44, alg8, algA, algB, algC, algD, algE, algF, algG, algI, algJ, algK, algL, algP/algR3, algQ, algR, algU, algW, algX, algZ, mucA, mucB, mucC*	*rhlA, rhlB*	*fptA, fpvA, pchA, pchB, pchC, pchD, pchE, pchF, pchG, pchH, pchI, pchR, pvdA, pvdE*	*phzM, phzS*	*aprA, lasA*	*toxA, plcH*	*lasI, rhlI*	*xcpP, xcpQ, xcpR, xcpS, xcpT, xcpU, xcpV, xcpW, xcpX, xcpY, xcpZ*
106	ST 1521	*waaA, waaC, waaF, waaG, waaP, wzy, wzz, chpA, chpB, chpC, chpD, chpE, fimT, fimU, fimV, pilA, pilB, pilD, pilE, pilF, pilG, pilH, pilI, pilK, pilM, pilN, pilO, pilP, pilQ, pilR, pilS, pilT, pilU, pilV, pilW, pilX pilY1, pilY2, pilC, xcpA/pilD*	*alg44, alg8, algA, algB, algC, algD, algE, algF, algG, algI, algJ, algK, algL, algQ, algR, algU, algW, algX, algZ, mucA, mucB, mucC*	*rhlA, rhlB*	*fptA, fpvA, pchA, pchB, pchC, pchD, pchE, pchF, pchG, pchH, pchI, pchR, pvdA, pvdD, pvdE*	*phzM, phzS*	*aprA, lasA*	*toxA, plcH*	*lasI, rhlI*	*xcpP, xcpQ, xcpR, xcpS, xcpT, xcpU, xcpV, xcpW, xcpX, xcpY, xcpZ*
114	ST 244	*waaA, waaC, waaF, waaG, waaP, wzy, wzz, chpA, chpB, chpC, chpD, chpE, fimT, fimU, fimV, pilA like, pilB, pilD, pilE, pilF, pilG, pilH, pilI, pilK, pilM, pilN, pilO, pilP, pilQ, pilR, pilS, pilT, pilU, pilV, pilW, pilX pilY1, pilY2, pilC, xcpA/pilD*	*alg44, alg8, algA, algB, algC, algD, algE, algF, algG, algI, algJ, algK, algL, algQ, algR, algU, algW, algX, algZ, mucA, mucB, mucC*	*rhlA, rhlB*	*fptA, fpvA, pchA, pchB, pchC, pchD, pchE, pchF, pchG, pchH, pchI, pchR, pvdA, pvdE*	*phzM, phzS*	*aprA, lasA*	*toxA, plcH*	*lasI, rhlI*	*xcpP, xcpQ, xcpR, xcpS, xcpT, xcpU, xcpV, xcpW like, xcpX, xcpY, xcpZ*
137	ST 3014	*waaA, waaC, waaF, waaG, waaP, wzy, wzz, chpA, chpB, chpC, chpD, chpE, fimT, fimU, fimV, pilA like, pilB, pilD, pilE, pilF, pilG, pilH, pilI, pilK, pilM, pilN, pilO, pilP, pilQ, pilR, pilS, pilT, pilU, pilV, pilW, pilX pilY1, pilY2, pilC, xcpA/pilD*	*alg44, alg8, algA, algB, algC, algD, algE, algF, algG, algI, algJ, algK, algL, algP/algR3, algQ, algR, algU, algW, algX, algZ, mucA, mucB, mucC*	*rhlA, rhlB*	*fptA, fpvA, pchA, pchB, pchC, pchD, pchE, pchF, pchG, pchH, pchI, pchR, pvdA, pvdE*	*phzM, phzS*	*aprA, lasA*	*toxA, plcH*	*lasI, rhlI*	*xcpP, xcpQ, xcpR, xcpS, xcpT, xcpU, xcpV, xcpW, xcpX, xcpY, xcpZ*
144	ST 245	*waaA, waaC, waaF, waaG, waaP, wzy, wzz, chpA, chpB, chpC, chpD, chpE, fimT, fimU, fimV, pilA, pilB, pilD, pilE, pilF, pilG, pilH, pilI, pilK, pilM, pilN, pilO, pilP, pilQ, pilR, pilS, pilT, pilU, pilV, pilW, pilX pilY1, pilY2, pilC like, xcpA/pilD*	*alg44, alg8, algA, algB, algC, algD, algE, algF, algG, algI, algJ, algK, algL, algQ, algR, algU, algW, algX, algZ, mucA, mucB, mucC*	*rhlA, rhlB*	*fptA, pchA, pchB, pchC, pchD, pchE, pchF, pchG, pchH, pchI, pchR, pvdA*	*phzM, phzS*	*aprA, lasA*	*toxA, plcH*	*lasI, rhlI*	*xcpP, xcpQ, xcpR, xcpS, xcpT, xcpU, xcpV, xcpW, xcpX, xcpY, xcpZ*
147	ST 245	*waaA, waaC, waaF, waaG, waaP, wzy, wzz, chpA, chpB, chpC, chpD, chpE, fimT, fimU, fimV, pilA, pilB, pilD, pilE, pilF, pilG, pilH, pilI, pilK, pilM, pilN, pilO, pilP, pilQ, pilR, pilS, pilT, pilU, pilV, pilW, pilX pilY1, pilY2, pilC like, xcpA/pilD*	*alg44, alg8, algA, algB, algC, algD, algE, algF, algG, algI, algJ, algK, algL, algQ, algR, algU, algW, algX, algZ, mucA, mucB, mucC*	*rhlA, rhlB*	*fptA, pchA, pchB, pchC, pchD, pchE, pchF, pchG, pchH, pchI, pchR, pvdA*	*phzM, phzS*	*aprA, lasA*	*toxA, plcH*	*lasI, rhlI*	*xcpP, xcpQ, xcpR, xcpS, xcpT, xcpU, xcpV, xcpW, xcpX, xcpY, xcpZ*
149	ST 381	*waaA, waaC, waaF, waaG, waaP, wzy, wzz, chpA, chpB, chpC, chpD, chpE, fimV, pilA, pilB, pilD, pilF, pilG, pilH, pilI, pilK, pilM, pilN, pilO, pilP, pilQ, pilR, pilS, pilT, pilU, pilC like, xcpA/pilD*	*alg44, alg8, algA, algB, algC, algD, algE, algF, algG, algI, algJ, algK, algL, algP/algR3, algQ, algR, algU, algW, algX, algZ, mucA, mucB, mucC*	*rhlA, rhlB*	*fptA, fpvA, pchA, pchB, pchC, pchD, pchE, pchF, pchG, pchH, pchI, pchR, pvdA, pvdE*	*phzM, phzS*	*aprA, lasA*	*toxA, plcH*	*lasI, rhlI*	*xcpP, xcpQ, xcpR, xcpS, xcpT, xcpU, xcpV, xcpW, xcpX, xcpY, xcpZ*
153	ST 704	*waaA, waaC, waaF, waaG, waaP, wzy, wzz, chpA, chpB, chpC, chpD, chpE, fimT, fimU, fimV, pilA, pilB, pilD, pilE, pilF, pilG, pilH, pilI, pilK, pilM, pilN, pilO, pilP, pilQ, pilR, pilS, pilT, pilU, pilV, pilW, pilX pilY1, pilY2, pilC like, xcpA/pilD*	*alg44, alg8, algA, algB, algC, algD, algE, algF, algG, algI, algJ, algK, algL, algQ, algR, algU, algW, algX, algZ, mucA, mucB, mucC*	*rhlA, rhlB*	*fptA, pchA, pchB, pchC, pchD, pchE, pchF, pchG, pchH, pchI, pchR, pvdA*	*phzM, phzS*	*aprA, lasA*	*plcH*	*lasI*	*xcpP, xcpQ, xcpR, xcpS, xcpT, xcpU, xcpV, xcpW, xcpX, xcpY, xcpZ*
154	ST 244	*waaA, waaC, waaF, waaG, waaP, wzy, wzz, chpA, chpB, chpC, chpD, chpE, fimT, fimU, fimV, pilA like, pilB, pilD, pilE, pilF, pilG, pilH, pilI, pilK, pilM, pilN, pilO, pilP, pilQ, pilR, pilS, pilT, pilU, pilV, pilW, pilX pilY1, pilY2, pilC, xcpA/pilD*	*alg44, alg8, algA, algB, algC, algD, algE, algF, algG, algI, algJ, algK, algL, algP/algR3, algQ, algR, algU, algW, algX, algZ, mucA, mucB, mucC*	*rhlA, rhlB*	*fptA, fpvA, pchA, pchB, pchC, pchD, pchE, pchF, pchG, pchH, pchI, pchR, pvdA, pvdE*	*phzM, phzS*	*aprA, lasA*	*toxA, plcH*	*lasI, rhlI*	*xcpP, xcpQ, xcpR, xcpS, xcpT, xcpU, xcpV, xcpW like, xcpX, xcpY, xcpZ*
157	ST 2616	*waaA, waaC, waaF, waaG, waaP, chpA, chpB, chpC, chpD, chpE, fimV, pilA, pilB, pilD, pilF, pilG, pilH, pilI, pilK, pilM, pilN, pilO, pilP, pilQ, pilR, pilS, pilT, pilU, pilC like, xcpA/pilD*	*alg44, alg8, algA, algB, algC, algD, algE, algF, algG, algI, algJ, algK, algL, algQ, algR, algU, algW, algX, algZ, mucA, mucB, mucC*	*rhlA, rhlB*	*fptA, fpvA, pchA, pchB, pchC, pchD, pchE, pchF, pchG, pchH, pchI, pchR, pvdA, pvdE*	*phzM, phzS*	*aprA, lasA*	*toxA, plcH*	*lasI, rhlI*	*xcpP, xcpQ, xcpR, xcpS, xcpT, xcpU, xcpV, xcpW, xcpX, xcpY, xcpZ*
160	ST 170	*waaA, waaC, waaF, waaG, waaP, chpA, chpB, chpC, chpD, chpE, fimV, pilA, pilB, pilD, pilF, pilG, pilH, pilI, pilK, pilM, pilN, pilO, pilP, pilQ, pilR, pilS, pilT, pilU, pilC like, xcpA/pilD*	*alg44, alg8, algA, algB, algC, algD, algE, algF, algG, algI, algJ, algK, algL, algP/algR3, algQ, algR, algU, algW, algX, algZ, mucA, mucB, mucC*	*rhlA, rhlB*	*fptA, pchA, pchB, pchC, pchD, pchE, pchF, pchG, pchH, pchI, pchR, pvdA*	*phzM, phzS*	*aprA, lasA*	*toxA, plcH*	*lasI, rhlI*	*xcpP, xcpQ, xcpR, xcpS, xcpT, xcpU, xcpV, xcpW, xcpX, xcpY, xcpZ*
162	ST 274	*waaA, waaC, waaF, waaG, waaP, chpA, chpB, chpC, chpD, chpE, fimV, pilA, pilB, pilD, pilF, pilG, pilH, pilI, pilK, pilM, pilN, pilO, pilP, pilQ, pilR, pilS, pilT, pilU, pilC like, xcpA/pilD*	*alg44, alg8, algA, algB, algC, algD, algE, algF, algG, algI, algJ, algK, algL, algP/algR3, algQ, algR, algU, algW, algX, algZ, mucA, mucB, mucC*	*rhlA, rhlB*	*fptA, pchA, pchB, pchC, pchD, pchE, pchF, pchG, pchH, pchI, pchR, pvdA*	*phzM, phzS*	*aprA, lasA*	*toxA, plcH*	*lasI, rhlI*	*xcpP, xcpQ, xcpR, xcpS, xcpT, xcpU, xcpV, xcpW, xcpX, xcpY, xcpZ*
180	ST 856	*waaA, waaC, waaF, waaG, waaP, chpA, chpB, chpC, chpD, chpE, fimT, fimU, fimV, pilA, pilB, pilD, pilE, pilF, pilG, pilH, pilI, pilK, pilM, pilN, pilO, pilP, pilQ, pilR, pilS, pilT, pilU, pilV, pilW, pilX pilY1, pilY2, pilC like, xcpA/pilD*	*alg44, alg8, algA, algB, algC, algD, algE, algF, algG, algI, algJ, algK, algL, algP/algR3, algQ, algR, algU, algW, algX, algZ, mucA, mucB, mucC*	*rhlA, rhlB*	*fptA, pchA, pchB, pchC, pchD, pchE, pchF, pchG, pchH, pchI, pchR, pvdA*	*phzM, phzS*	*aprA, lasA*	*toxA, plcH*	*lasI, rhlI*	*xcpP, xcpQ, xcpR, xcpS, xcpT, xcpU, xcpV, xcpW, xcpX, xcpY, xcpZ*
183	ST 244	*waaA, waaC, waaF, waaG, waaP, wzy, wzz, chpA, chpB, chpC, chpD, chpE, fimT, fimU, fimV, pilA like, pilB, pilD, pilE, pilF, pilG, pilH, pilI, pilK, pilM, pilN, pilO, pilP, pilQ, pilR, pilS, pilT, pilU, pilV, pilW, pilX pilY1, pilY2, pilC, xcpA/pilD*	*alg44, alg8, algA, algB, algC, algD, algE, algF, algG, algI, algJ, algK, algL, algQ, algR, algU, algW, algX, algZ, mucA, mucB, mucC*	*rhlA, rhlB*	*fptA, fpvA, pchA, pchB, pchC, pchD, pchE, pchF, pchG, pchH, pchI, pchR, pvdA, pvdE*	*phzM, phzS*	*aprA, lasA*	*toxA, plcH*	*lasI, rhlI*	*xcpP, xcpQ, xcpR, xcpS, xcpT, xcpU, xcpV, xcpW like, xcpX, xcpY, xcpZ*
186	ST 3588	*waaA, waaC, waaF, waaG, waaP, chpA, chpB, chpC, chpD, chpE, fimT, fimU, fimV, pilA like, pilB, pilD, pilE, pilF, pilG, pilH, pilI, pilK, pilM, pilN, pilO, pilP, pilQ, pilR, pilS, pilT, pilU, pilV, pilW, pilX pilY1, pilY2, pilC, xcpA/pilD*	*alg44, alg8, algA, algB, algC, algD, algE, algF, algG, algI, algJ, algK, algL, algQ, algR, algU, algW, algX, algZ, mucA, mucB, mucC*	*rhlA, rhlB*	*fptA, pchA, pchB, pchC, pchD, pchE, pchF, pchG, pchH, pchI, pchR, pvdA*	*phzM, phzS*	*aprA, lasA*	*plcH*	*lasI, rhlI*	*xcpP, xcpQ, xcpR, xcpS, xcpT, xcpU, xcpV, xcpW, xcpX, xcpY, xcpZ*
190	ST 871	*waaA, waaC, waaF, waaG, waaP, chpA, chpB, chpC, chpD, chpE, fimT, fimU, fimV, pilA, pilB, pilD, pilE, pilF, pilG, pilH, pilI, pilK, pilM, pilN, pilO, pilP, pilQ, pilR, pilS, pilT, pilU, pilV, pilW, pilX pilY1, pilY2, pilC like, xcpA/pilD*	*alg44, alg8, algA, algB, algC, algD, algE, algF, algG, algI, algJ, algK, algL, algP/algR3, algQ, algR, algU, algW, algX, algZ, mucA, mucB, mucC*	*rhlA, rhlB*	*fptA, pchA, pchB, pchC, pchD, pchE, pchF, pchG, pchH, pchI, pchR, pvdA*	*phzM, phzS*	*aprA, lasA*	*toxA, plcH*	*lasI, rhlI*	*xcpP, xcpQ, xcpR, xcpS, xcpT, xcpU, xcpV, xcpW, xcpX, xcpY, xcpZ*
195	ST 988	*waaA, waaC, waaF, waaG, waaP, chpA, chpB, chpC, chpD, chpE, fimT, fimU, fimV, pilA, pilB, pilD, pilE, pilF, pilG, pilH, pilI, pilK, pilM, pilN, pilO, pilP, pilQ, pilR, pilS, pilT, pilU, pilV, pilW, pilX pilY1, pilY2, pilC like, xcpA/pilD*	*alg44, alg8, algA, algB, algC, algD, algE, algF, algG, algI, algJ, algK, algL, algP/algR3, algQ, algR, algU, algW, algX, algZ, mucA, mucB, mucC*	*rhlA, rhlB*	*fptA, pchA, pchB, pchC, pchD, pchE, pchF, pchG, pchH, pchI, pchR, pvdA*	*phzM, phzS*	*aprA, lasA*	*toxA, plcH*	*lasI, rhlI*	*xcpP, xcpQ, xcpR, xcpS, xcpT, xcpU, xcpV, xcpW, xcpX, xcpY, xcpZ*
196	ST 2475	*waaA, waaC, waaF, waaG, waaP, wzy, wzz, chpA, chpB, chpC, chpD, chpE, fimT, fimU, fimV, pilA, pilB, pilD, pilE, pilF, pilG, pilH, pilI, pilK, pilM, pilN, pilO, pilP, pilQ, pilR, pilS, pilT, pilU, pilV, pilW, pilX pilY1, pilY2, pilC, xcpA/pilD*	*alg44, alg8, algA, algB, algC, algD, algE, algF, algG, algI, algJ, algK, algL, algP/algR3, algQ, algR, algU, algW, algX, algZ, mucA, mucB, mucC*	*rhlA, rhlB*	*fptA, fpvA pchA, pchB, pchC, pchD, pchE, pchF, pchG, pchH, pchI, pchR, pvdA, pvdE*	*phzM, phzS*	*aprA, lasA*	*toxA, plcH*	*lasI, rhlI*	*xcpP, xcpQ, xcpR, xcpS, xcpT, xcpU, xcpV, xcpW, xcpX, xcpY, xcpZ*
198	ST 2476	*waaA, waaC, waaF, waaG, waaP, chpA, chpB, chpC, chpD, chpE, fimV, pilB, pilD, pilF, pilG, pilH, pilI, pilK, pilM, pilN, pilO, pilP, pilQ, pilR, pilS, pilT, pilU, pilC like, xcpA/pilD*	*alg44, alg8, algA, algB, algC, algD, algE, algF, algG, algI, algJ, algK, algL, algP/algR3, algQ, algR, algU, algW, algX, algZ, mucA, mucB, mucC*	*rhlA, rhlB*	*fptA, pchA, pchB, pchC, pchD, pchE, pchF, pchG, pchH, pchI, pchR, pvdA*	*phzM, phzS*	*aprA, lasA*	*toxA, plcH*	*lasI, rhlI*	*xcpP, xcpQ, xcpR, xcpS, xcpT, xcpU, xcpV, xcpW, xcpX, xcpY, xcpZ*
204	ST 639	*waaA, waaC, waaF, waaG, waaP, chpA, chpB, chpC, chpD, chpE, fimT, fimU, fimV, pilB, pilD, pilE, pilF, pilG, pilH, pilI, pilK, pilM, pilN, pilO, pilP, pilQ, pilR, pilS, pilT, pilU, pilV, pilW, pilX pilY1, pilY2, pilC like, xcpA/pilD*	*alg44, alg8, algA, algB, algC, algD, algE, algF, algG, algI, algJ, algK, algL, algQ, algR, algU, algW, algX, algZ, mucA, mucB, mucC*	*rhlA, rhlB*	*fptA, pchA, pchB, pchC, pchD, pchE, pchF, pchG, pchH, pchI, pchR, pvdA*	*phzM, phzS*	*aprA, lasA*	*toxA, plcH*	*rhlI*	*xcpP, xcpQ, xcpR, xcpS, xcpT, xcpU, xcpV, xcpW, xcpX, xcpY, xcpZ*
208	ST 132	*waaA, waaC, waaF, waaG, waaP, chpA, chpB, chpC, chpD, chpE, fimT, fimU, fimV, pilA like, pilB, pilD, pilE, pilF, pilG, pilH, pilI, pilK, pilM, pilN, pilO, pilP, pilQ, pilR, pilS, pilT, pilU, pilV, pilW, pilX pilY1, pilY2, pilC, xcpA/pilD*	*alg44, alg8, algA, algB, algC, algD, algE, algF, algG, algI, algJ, algK, algL, algQ, algR, algU, algW, algX, algZ, mucA, mucB, mucC*	*rhlA, rhlB*	*fptA, pchA, pchB, pchC, pchD, pchE, pchF, pchG, pchH, pchI, pchR, pvdA*	*phzM, phzS*	*aprA, lasA*	*toxA, plcH*	*lasI, rhlI*	*xcpP, xcpQ, xcpR, xcpS, xcpT, xcpU, xcpV, xcpW, xcpX, xcpY, xcpZ*
218	ST 856	*waaA, waaC, waaF, waaG, waaP, chpA, chpB, chpC, chpD, chpE, fimT, fimU, fimV, pilB, pilD, pilE, pilF, pilG, pilH, pilI, pilK, pilM, pilN, pilO, pilP, pilQ, pilR, pilS, pilT, pilU, pilV, pilW, pilX pilY1, pilY2, pilC like, xcpA/pilD*	*alg44, alg8, algA, algB, algC, algD, algE, algF, algG, algI, algJ, algK, algL, algQ, algR, algU, algW, algX, algZ, mucA, mucB, mucC*	*rhlA, rhlB*	*fptA, pchA, pchB, pchC, pchD, pchE, pchF, pchG, pchH, pchI, pchR, pvdA*	*phzM, phzS*	*aprA, lasA*	*toxA, plcH*	*lasI, rhlI*	*xcpP, xcpQ, xcpR, xcpS, xcpT, xcpU, xcpV, xcpW, xcpX, xcpY, xcpZ*
229	ST 270	*waaA, waaC, waaF, waaG, waaP, wzy, wzz, chpA, chpB, chpC, chpD, chpE, fimT, fimU, fimV, pilB, pilD, pilE, pilF, pilG, pilH, pilI, pilK, pilM, pilN, pilO, pilP, pilQ, pilR, pilS, pilT, pilU, pilV, pilW, pilX pilY1, pilY2, pilC like, xcpA/pilD*	*alg44, alg8, algA, algB, algC, algD, algE, algF, algG, algI, algJ, algK, algL, algP/algR3, algQ, algR, algU, algW, algX, algZ, mucA, mucB, mucC*	*rhlA, rhlB*	*fptA, fpvA pchA, pchB, pchC, pchD, pchE, pchF, pchG, pchH, pchI, pchR, pvdA, pvdD, pvdE*	*phzM, phzS*	*aprA, lasA*	*toxA, plcH*	*lasI, rhlI*	*xcpP, xcpQ, xcpR, xcpS, xcpT, xcpU, xcpV, xcpW, xcpX, xcpY, xcpZ*
233	ST 3227	*waaA, waaC, waaF, waaG, waaP, chpA, chpB, chpC, chpD, chpE, fimV, pilB, pilD, pilF, pilG, pilH, pilI, pilK, pilM, pilN, pilO, pilP, pilQ, pilR, pilS, pilT, pilU, pilC like, xcpA/pilD*	*alg44, alg8, algA, algB, algC, algD, algE, algF, algG, algI, algJ, algK, algL, algP/algR3, algQ, algR, algU, algW, algX, algZ, mucA, mucB, mucC*	*rhlA, rhlB*	*fptA, pchA, pchB, pchC, pchD, pchE, pchF, pchG, pchH, pchI, pchR, pvdA*	*phzM, phzS*	*aprA, lasA*	*toxA, plcH*	*lasI, rhlI*	*xcpP, xcpQ, xcpR, xcpS, xcpT, xcpU, xcpV, xcpW, xcpX, xcpY, xcpZ*
236	ST 266	*waaA, waaC, waaF, waaG, waaP, chpA, chpB, chpC, chpD, chpE, fimT, fimU, fimV, pilB, pilD, pilE, pilF, pilG, pilH, pilI, pilK, pilM, pilN, pilO, pilP, pilQ, pilR, pilS, pilT, pilU, pilV, pilW, pilX pilY1, pilY2, pilC like, xcpA/pilD*	*alg44, alg8, algA, algB, algC, algD, algE, algF, algG, algI, algJ, algK, algL, algQ, algR, algU, algW, algX, algZ, mucA, mucB, mucC*	*rhlA, rhlB*	*fptA, pchA, pchB, pchC, pchD, pchE, pchF, pchG, pchH, pchI, pchR, pvdA*	*phzM, phzS*	*aprA, lasA*	*toxA, plcH*	*lasI, rhlI*	*xcpP, xcpQ, xcpR, xcpS, xcpT, xcpU, xcpV, xcpW, xcpX, xcpY, xcpZ*
238	ST 3589	*waaA, waaC, waaF, waaG, waaP, chpA, chpB, chpC, chpD, chpE, fimT, fimU, fimV, pilA, pilB, pilD, pilE, pilF, pilG, pilH, pilI, pilK, pilM, pilN, pilO, pilP, pilQ, pilR, pilS, pilT, pilU, pilV, pilW, pilX pilY1, pilY2, pilC like, xcpA/pilD*	*alg44, alg8, algA, algB, algC, algD, algE, algF, algG, algI, algJ, algK, algL, algQ, algR, algU, algW, algX, algZ, mucA, mucB, mucC*	*rhlA, rhlB*	*fptA, pchA, pchB, pchC, pchD, pchE, pchF, pchG, pchH, pchI, pchR, pvdA*	*phzM, phzS*	*aprA, lasA*	*plcH*	*lasI, rhlI*	*xcpP, xcpQ, xcpS, xcpT, xcpU, xcpV, xcpW, xcpX, xcpY, xcpZ*
242	ST 3590	*waaA, waaC, waaF, waaG, waaP, chpA, chpB, chpC, chpD, chpE, fimT, fimU, fimV, pilB, pilD, pilE, pilF, pilG, pilH, pilI, pilK, pilM, pilN, pilO, pilP, pilQ, pilR, pilS, pilT, pilU, pilV, pilW, pilX pilY1, pilY2, pilC like, xcpA/pilD*	*alg44, alg8, algA, algB, algC, algD, algE, algF, algG, algI, algJ, algK, algL, algP/algR3 like, algQ, algR, algU, algW, algX, algZ, mucA, mucB, mucC*	*rhlA, rhlB*	*fptA, fpvA pchA, pchB, pchC, pchD, pchE, pchF, pchG, pchH, pchI, pchR, pvdA, pvdE*	*phzM, phzS*	*aprA, lasA*	*toxA, plcH*	*lasI, rhlI*	*xcpP, xcpQ, xcpS, xcpT, xcpU, xcpV, xcpW, xcpX, xcpY, xcpZ*
243	ST 3590	*waaA, waaC, waaF, waaG, waaP, chpA, chpB, chpC, chpD, chpE, fimT, fimU, fimV, pilB, pilD, pilE, pilF, pilG, pilH, pilI, pilK, pilM, pilN, pilO, pilP, pilQ, pilR, pilS, pilT, pilU, pilV, pilW, pilX pilY1, pilY2, pilC like, xcpA/pilD*	*alg44, alg8, algA, algB, algC, algD, algE, algF, algG, algI, algJ, algK, algL, algP/algR3, algQ, algR, algU, algW, algX, algZ, mucA, mucB, mucC*	*rhlA, rhlB*	*fptA, fpvA pchA, pchB, pchC, pchD, pchE, pchF, pchG, pchH, pchI, pchR, pvdA, pvdE*	*phzM, phzS*	*aprA, lasA*	*toxA, plcH*	*lasI, rhlI*	*xcpP, xcpQ, xcpS, xcpT, xcpU, xcpV, xcpW, xcpX, xcpY, xcpZ*
272	ST 2033	*waaA, waaC, waaF, waaG, waaP, chpA, chpB, chpC, chpD, chpE, fimT, fimU, fimV, pilA, pilB, pilD, pilE, pilF, pilG, pilH, pilI, pilK, pilM, pilN, pilO, pilP, pilQ, pilR, pilS, pilT, pilU, pilV, pilW, pilX pilY1, pilY2, pilC, xcpA/pilD*	*alg44, alg8, algA, algB, algC, algD, algE, algF, algG, algI, algJ, algK, algL, algQ, algR, algU, algW, algX, algZ, mucA, mucB, mucC*	*rhlA, rhlB*	*fptA, pchA, pchB, pchC, pchD, pchE, pchF, pchG, pchH, pchI, pchR, pvdA*	*phzM, phzS*	*aprA, lasA*	*toxA, plcH*	*lasI, rhlI*	*xcpP, xcpQ, xcpR, xcpS, xcpT, xcpU, xcpV, xcpW, xcpX, xcpY, xcpZ*
274	ST 2033	*waaA, waaC, waaF, waaG, waaP, chpA, chpB, chpC, chpD, chpE, fimT, fimU, fimV, pilA, pilB, pilD, pilE, pilF, pilG, pilH, pilI, pilK, pilM, pilN, pilO, pilP, pilQ, pilR, pilS, pilT, pilU, pilV, pilW, pilX pilY1, pilY2, pilC, xcpA/pilD*	*alg44, alg8, algA, algB, algC, algD, algE, algF, algG, algI, algJ, algK, algL, algQ, algR, algU, algW, algX, algZ, mucA, mucB, mucC*	*rhlA, rhlB*	*fptA, pchA, pchB, pchC, pchD, pchE, pchF, pchG, pchH, pchI, pchR, pvdA*	*phzM, phzS*	*aprA, lasA*	*toxA, plcH*	*lasI, rhlI*	*xcpP, xcpQ, xcpR, xcpS, xcpT, xcpU, xcpV, xcpW, xcpX, xcpY, xcpZ*
278	ST 988	*waaA, waaC, waaF, waaG, waaP, chpA, chpB, chpC, chpD, chpE, fimT, fimU, fimV, pilB, pilD, pilE, pilF, pilG, pilH, pilI, pilK, pilM, pilN, pilO, pilP, pilQ, pilR, pilS, pilT, pilU, pilV, pilW, pilX pilY1, pilY2, pilC like, xcpA/pilD*	*alg44, alg8, algA, algB, algC, algD, algE, algF, algG, algI, algJ, algK, algL, algQ, algR, algU, algW, algX, algZ, mucA, mucB, mucC*	*rhlA, rhlB*	*fptA, pchA, pchB, pchC, pchD, pchE, pchF, pchG, pchH, pchI, pchR, pvdA*	*phzM, phzS*	*aprA, lasA*	*toxA, plcH*	*lasI, rhlI*	*xcpP, xcpQ, xcpR, xcpS, xcpT, xcpU, xcpV, xcpW, xcpX, xcpY, xcpZ*
282	ST 554	*waaA, waaC, waaF, waaG, waaP, wzy, wzz, chpA, chpB, chpC, chpD, chpE, fimT, fimU, fimV, pilA like, pilB, pilD, pilE, pilF, pilG, pilH, pilI, pilK, pilM, pilN, pilO, pilP, pilQ, pilR, pilS, pilT, pilU, pilV, pilW, pilX pilY1, pilY2, pilC, xcpA/pilD*	*alg44, alg8, algA, algB, algC, algD, algE, algF, algG, algI, algJ, algK, algL, algQ, algR, algU, algW, algX, algZ, mucA, mucB, mucC*	*rhlA, rhlB*	*fptA, pchA, pchB, pchC, pchD, pchE, pchF, pchG, pchH, pchI, pchR, pvdA*	*phzM, phzS*	*aprA, lasA*	*toxA, plcH*	*lasI, rhlI*	*xcpP, xcpQ, xcpR, xcpS, xcpT, xcpU, xcpV, xcpW, xcpX, xcpY, xcpZ*
285	ST 554	*waaA, waaC, waaF, waaG, waaP, wzy, wzz, chpA, chpB, chpC, chpD, chpE, fimT, fimU, fimV, pilA like, pilB, pilD, pilE, pilF, pilG, pilH, pilI, pilK, pilM, pilN, pilO, pilP, pilQ, pilR, pilS, pilT, pilU, pilV, pilW, pilX pilY1, pilY2, pilC, xcpA/pilD*	*alg44, alg8, algA, algB, algC, algD, algE, algF, algG, algI, algJ, algK, algL, algQ, algR, algU, algW, algX, algZ, mucA, mucB, mucC*	*rhlA, rhlB*	*fptA, pchA, pchB, pchC, pchD, pchE, pchF, pchG, pchH, pchI, pchR, pvdA*	*phzM, phzS*	*aprA, lasA*	*toxA, plcH*	*lasI, rhlI*	*xcpP, xcpQ, xcpR, xcpS, xcpT, xcpU, xcpV, xcpW, xcpX, xcpY, xcpZ*
289	ST 1485	*waaA, waaC, waaF, waaG, waaP, chpA, chpB, chpC, chpD, chpE, fimT, fimU, fimV, pilB, pilD, pilE, pilF, pilG, pilH, pilI, pilK, pilM, pilN, pilO, pilP, pilQ, pilR, pilS, pilT, pilU, pilV, pilW, pilX pilY1, pilY2, pilC like, xcpA/pilD*	*alg44, alg8, algA, algB, algC, algD, algE, algF, algG, algI, algJ, algK, algL, algP/algR3, algQ, algR, algU, algW, algX, algZ, mucA, mucB, mucC*	*rhlA, rhlB*	*fptA, fpvA pchA, pchB, pchC, pchD, pchE, pchF, pchG, pchH, pchI, pchR, pvdA, pvdD, pvdE*	*phzM, phzS*	*aprA, lasA*	*toxA, plcH*	*lasI, rhlI*	*xcpP, xcpQ, xcpR, xcpS, xcpT, xcpU, xcpV, xcpW, xcpX, xcpY, xcpZ*
290	ST 1485	*waaA, waaC, waaF, waaG, waaP, chpA, chpB, chpC, chpD, chpE, fimT, fimU, fimV, pilB, pilD, pilE, pilF, pilG, pilH, pilI, pilK, pilM, pilN, pilO, pilP, pilQ, pilR, pilS, pilT, pilU, pilV, pilW, pilX pilY1, pilY2, pilC like, xcpA/pilD*	*alg44, alg8, algA, algB, algC, algD, algE, algF, algG, algI, algJ, algK, algL, algP/algR3, algQ, algR, algU, algW, algX, algZ, mucA, mucB, mucC*	*rhlA, rhlB*	*fptA, fpvA pchA, pchB, pchC, pchD, pchE, pchF, pchG, pchH, pchI, pchR, pvdA, pvdD, pvdE*	*phzM, phzS*	*aprA, lasA*	*toxA, plcH*	*lasI, rhlI*	*xcpP, xcpQ, xcpR, xcpS, xcpT, xcpU, xcpV, xcpW, xcpX, xcpY, xcpZ*
296	ST 235	*waaA, waaC, waaF, waaG, waaP, chpA, chpB, chpC, chpD, chpE, fimT, fimU, fimV, pilB, pilD, pilE, pilF, pilG, pilH, pilI, pilK, pilM, pilN, pilO, pilP, pilQ, pilR, pilS, pilT, pilU, pilW, pilX pilY1, pilY2, pilC, xcpA/pilD*	*alg44, alg8, algA, algB, algC, algD, algE, algF, algG, algI, algJ, algK, algL, algQ, algR, algU, algW, algX, algZ, mucA, mucB, mucC*	*rhlA, rhlB*	*fptA, pchA, pchB, pchC, pchD, pchE, pchF, pchG, pchH, pchI, pchR, pvdA*	*phzM, phzS*	*aprA, lasA*	*toxA, plcH*	*lasI, rhlI*	*xcpP, xcpQ, xcpR, xcpS, xcpT, xcpU, xcpV, xcpW like, xcpX, xcpY, xcpZ*
298	ST 3227	*waaA, waaC, waaF, waaG, waaP, chpA, chpB, chpC, chpD, chpE, fimV, pilB, pilD, pilF, pilG, pilH, pilI, pilK, pilM, pilN, pilO, pilP, pilQ, pilR, pilS, pilT, pilU, pilV, pilC, xcpA/pilD*	*alg44, alg8, algA, algB, algC, algD, algE, algF, algG, algI, algJ, algK, algL, algQ, algR, algU, algW, algX, algZ, mucA, mucB, mucC*	*rhlA, rhlB*	*fptA, pchA, pchB, pchC, pchD, pchE, pchF, pchG, pchH, pchI, pchR, pvdA*	*phzM, phzS*	*aprA, lasA*	*toxA, plcH*	*lasI, rhlI*	*xcpP, xcpQ, xcpR, xcpS, xcpT, xcpU, xcpV, xcpW, xcpX, xcpY, xcpZ*
301	ST 3593	*waaA, waaC, waaF, waaG, waaP, wzy, wzz, chpA, chpB, chpC, chpD, chpE, fimT, fimU, fimV, pilB, pilD, pilE, pilF, pilG, pilH, pilI, pilK, pilM, pilN, pilO, pilP, pilQ, pilR, pilS, pilT, pilU, pilV, pilW, pilX pilY1, pilY2, pilC, xcpA/pilD*	*alg44, alg8, algA, algB, algC, algD, algE, algF, algG, algI, algJ, algK, algL, algP/algR3, algQ, algR, algU, algW, algX, algZ, mucA, mucB, mucC*	*rhlA, rhlB*	*fptA, fpvA pchA, pchB, pchC, pchD, pchE, pchF, pchG, pchH, pchI, pchR, pvdA, pvdE*	*phzM, phzS*	*aprA, lasA*	*plcH*	*lasI, rhlI*	*xcpP, xcpQ, xcpR, xcpS, xcpT, xcpU, xcpV, xcpW, xcpX, xcpY, xcpZ*
302	ST 1755	*waaA, waaC, waaF, waaG, waaP, wzy, wzz, chpA, chpB, chpC, chpD, chpE, fimT, fimU, fimV, pilB, pilD, pilE, pilF, pilG, pilH, pilI, pilK, pilM, pilN, pilO, pilP, pilQ, pilR, pilS, pilT, pilU, pilV, pilW, pilX pilY1, pilY2, pilC like, xcpA/pilD*	*alg44, alg8, algA, algB, algC, algD, algE, algF, algG, algI, algJ, algK, algL, algQ, algR, algU, algW, algX, algZ, mucA, mucB, mucC*	*rhlA, rhlB*	*fptA, fpvA pchA, pchB, pchC, pchD, pchE, pchF, pchG, pchH, pchI, pchR, pvdA, pvdE*	*phzM, phzS*	*aprA, lasA*	*toxA, plcH*	*lasI, rhlI*	*xcpP, xcpQ, xcpR, xcpS, xcpT, xcpU, xcpV, xcpW, xcpX, xcpY, xcpZ*
309	ST 3592	*waaA, waaC, waaF, waaG, waaP, wzy, wzz, chpA, chpB, chpC, chpD, chpE, fimT, fimU, fimV, pilB, pilD, pilE, pilF, pilG, pilH, pilI, pilK, pilM, pilN, pilO, pilP, pilQ, pilR, pilS, pilT, pilU, pilV, pilW, pilX pilY1, pilY2, pilC, xcpA/pilD*	*alg44, alg8, algA, algB, algC, algD, algE, algF, algG, algI, algJ, algK, algL, algQ, algR, algU, algW, algX, algZ, mucA, mucB, mucC*	*rhlA, rhlB*	*fptA, fpvA pchA, pchB, pchC, pchD, pchE, pchF, pchG, pchH, pchI, pchR, pvdA, pvdE*	*phzM, phzS*	*aprA, lasA*	*plcH*	*lasI, rhlI*	*xcpP, xcpQ, xcpS, xcpT, xcpU, xcpV, xcpW, xcpX, xcpY, xcpZ*
310	ST 532	*waaA, waaC, waaF, waaG, waaP, chpA, chpB, chpC, chpD, chpE, fimV, pilB, pilD, pilF, pilG, pilH, pilI, pilK, pilM, pilN, pilO, pilP, pilQ, pilR, pilS, pilT, pilU, pilC like, xcpA/pilD*	*alg44, alg8, algA, algB, algC, algD, algE, algF, algG, algI, algJ, algK, algL, algQ, algR, algU, algW, algX, algZ, mucA, mucB, mucC*	*rhlA, rhlB*	*fptA, fpvA pchA, pchB, pchC, pchD, pchE, pchF, pchG, pchH, pchI, pchR, pvdA*	*phzM, phzS*	*aprA lasA*	*toxA, plcH*	*lasI, rhlI*	*xcpP, xcpQ, xcpR, xcpS, xcpT, xcpU, xcpV, xcpW, xcpX, xcpY, xcpZ*
312	ST 381	*waaA, waaC, waaF, waaG, waaP, wzy, wzz, chpA, chpB, chpC, chpD, chpE, fimV, pilB, pilD, pilF, pilG, pilH, pilI, pilK, pilM, pilN, pilO, pilP, pilQ, pilR, pilS, pilT, pilU, pilC like, xcpA/pilD*	*alg44, alg8, algA, algB, algC, algD, algE, algF, algG, algI, algJ, algK, algL, algP/algR3, algQ, algR, algU, algW, algX, algZ, mucA, mucB, mucC*	*rhlA, rhlB*	*fptA, pchA, pchB, pchC, pchD, pchE, pchF, pchG, pchH, pchI, pchR, pvdA, pvdD, pvdE*	*phzM, phzS*	*aprA, lasA*	*toxA, plcH*	*lasI, rhlI*	*xcpP, xcpQ, xcpR, xcpS, xcpT, xcpU, xcpV, xcpW, xcpX, xcpY, xcpZ*

**Table A10:** Analysis of virulence determinants, ordered by strain and MLST type, of the assessed *K. pneumoniae* isolates. ST = Sequence type.

Sample ID	ST-Type	Pathogenicity Factor Groups									
		Adherence	Biofilm Formation	Efflux Pump	Immune Evasion	Iron Uptake	Nutritional Factor	Regulation	Secretion System	Serum Resistance	Toxin
044	ST 327	*fimA, fimB, fimC, fimD, fimE, fimF, fimG, fimH, fimI, fimK*	*mrkA, mrkB, mrkC, mrkD, mrkF, mrkH, mrkI, mrkJ*	*acrA, acrB*	*cpsACP, galF, gnd, ugd, wza like, wzi*	*entA, entB, entC, entD, entE, entF, fepA, fepB, fepC, fepD, fepG, fes, ybdA, iroE like*		*rcsA, rcsB*	*impA/tssA like, sciN/tssJ, tssF, tssG, vasE/tssK, vgrG/tssI, vipA/tssB, vipB/tssC*	*glf, wbbM, wbbN, wbbO, wzm, wzt*	
060	ST 5379	*fimA, fimB, fimC, fimD, fimE, fimF, fimG, fimH, fimI, fimK*	*mrkA, mrkB, mrkC, mrkD, mrkF, mrkH, mrkI, mrkJ*	*acrA, acrB*	*cpsACP, galF, gnd, ugd, wza like, wzi*	*entA, entB, entC, entE, entF, fepA, fepB, fepC, fepD, fepG, fes, ybdA, iroE*		*rcsA, rcsB*	*impA/tssA, sciN/tssJ, tssF, tssG, vasE/tssK, vgrG/tssI, vipA/tssB, vipB/tssC*		
073	ST 39	*fimA, fimB, fimC, fimD, fimE, fimF, fimG, fimH, fimI, fimK*	*mrkA, mrkB, mrkC, mrkD, mrkF, mrkH, mrkI, mrkJ*	*acrA, acrB*	*cpsACP, galF, gnd, ugd, wza like, wzi*	*entA, entB, entC, entE, entF, fepA, fepB, fepC, fepD, fepG, fes, ybdA, iroE, irp1, irp2, ybtA, ybtE, ybtP, ybtQ, ybtS, ybtT, ybtU, ybtX*		*rcsA, rcsB*	*impA/tssA, sciN/tssJ, tle1, tli1, tssF, tssG, vasE/tssK, vgrG/tssI, vipA/tssB, vipB/tssC*	*glf, wbbM, wbbN, wbbO, wzm, wzt*	
100	ST 152	*fimA, fimB, fimC, fimD, fimE, fimF, fimG, fimH, fimI, fimK*	*mrkA, mrkB, mrkC, mrkD, mrkF, mrkH, mrkI, mrkJ*	*acrA, acrB*	*cpsACP, galF, gnd, ugd, wza like, wzi*	*entA, entB, entC, entD, entE, entF, fepA, fepB, fepC, fepD, fepG, fes, ybdA, iroE, irp1, irp2, ybtA, ybtE, ybtP, ybtQ, ybtS, ybtT, ybtU, ybtX*		*rcsA, rcsB*	*impA/tssA like, sciN/tssJ, tssF, tssG*		
102	ST 514	*fimA, fimB, fimC, fimD, fimE, fimF, fimG, fimH, fimI, fimK*	*mrkA, mrkB, mrkC, mrkD, mrkF, mrkI, mrkJ*	*acrA, acrB*	*cpsACP, galF, gnd, manB, manC, ugd, wza, wzi*	*entA, entB, entC, entE, entF, fepA, fepB, fepC, fepD, fepG, fes, ybdA, iroE*		*rcsA, rcsB*	*impA/tssA like, sciN/tssJ, tssF, tssG, vasE/tssK, vgrG/tssI, vipA/tssB, vipB/tssC*	*glf, wbbM, wbbN, wbbO, wzm, wzt*	
124	ST 399	*fimA, fimB, fimC, fimD, fimE, fimF, fimG, fimH, fimI, fimK*	*mrkF, mrkH, mrkJ*	*acrA, acrB*	*cpsACP, galF, ugd, wza like, wzi*	*entA, entB, entC, entE, entF, fepA, fepB, fepC, fepD, fepG, fes, ybdA, iroE*		*rcsA, rcsB*		*glf, wbbM, wbbN, wbbO, wzm, wzt*	
146	ST 4	*fimA, fimC, fimD, fimE, fimF, fimG, fimH, fimI, fimK*	*mrkA, mrkB, mrkC, mrkD, mrkF, mrkH, mrkI, mrkJ*	*acrA, acrB*	*cpsACP, galF, gnd, manB, manC, ugd, wza, wzi*	*entA, entB, entC, entE, entF, fepA, fepB, fepC, fepD, fepG, fes, ybdA, iroE, irp1, irp2, ybtA, ybtE, ybtP, ybtQ, ybtS, ybtT, ybtU, ybtX*		*rcsA, rcsB*	*impA/tssA like, sciN/tssJ, tssF, tssG, vasE/tssK, vgrG/tssI, vipA/tssB, vipB/tssC*	*glf, wbbM, wbbN, wbbO, wzm, wzt*	
177	ST 17	*fimA, fimB, fimC, fimD, fimE, fimF, fimG, fimH, fimI, fimK*	*mrkA, mrkD, mrkF, mrkH, mrkI, mrkJ*	*acrA, acrB*	*cpsACP, galF, gnd, manB, manC, ugd, wza like, wzi*	*entA, entB, entC, entD, entE, entF, fepA, fepB, fepC, fepD, fepG, fes, ybdA, iroE, ybtA, ybtE, ybtP, ybtQ, ybtS, ybtT, ybtU, ybtX*		*rcsA, rcsB*	*impA/tssA, sciN/tssJ, tssF, tssG, vasE/tssK, vgrG/tssI, vipA/tssB, vipB/tssC*	*glf, wbbM, wbbN, wbbO, wzm, wzt*	
181	ST 5380	*fimA, fimB, fimC, fimD, fimE, fimF, fimG, fimH, fimI, fimK*	*mrkA, mrkB, mrkC, mrkD, mrkF, mrkH, mrkI, mrkJ*	*acrA, acrB*	*cpsACP, galF, gmd like, gnd, manB, manC, ugd, wza like, wzi*	*entA, entB, entC, entE, entF, fepA, fepB, fepC, fepD, fepG, fes, ybdA, iroE*		*rcsA, rcsB,*	*impA/tssA, sciN/tssJ, tle1, tli1, tssF, tssG, vasE/tssK, vgrG/tssI, vipA/tssB, vipB/tssC*	*glf, wbbM, wbbN, wbbO, wzm, wzt*	
184	ST 5381	*fimA, fimB, fimC, fimD, fimE, fimF, fimG, fimH, fimI, fimK*	*mrkA, mrkB, mrkC, mrkD, mrkF, mrkH, mrkI, mrkJ*	*acrA, acrB*	*cpsACP, galF, gnd, ugd, wza like, wzi*	*entA, entB, entC, entD like, entE, entF, fepA, fepB, fepC, fepD, fepG, fes, ybdA, iroE like*		*rcsA, rcsB*	*impA/tssA, sciN/tssJ, tssF, tssG, vasE/tssK, vgrG/tssI, vipA/tssB, vipB/tssC*		
199	ST 17	*fimA, fimB, fimC, fimD, fimE, fimF, fimG, fimH, fimI, fimK*	*mrkA, mrkB, mrkC, mrkD, mrkF, mrkH, mrkI, mrkJ*	*acrA, acrB*	*cpsACP, galF, gnd, manB like, manC, ugd, wza, wzi*	*entA, entB, entC, entE, entF, fepA, fepB, fepC, fepD, fepG, fes, ybdA, iroE, irp1, irp2, ybtA, ybtE, ybtP, ybtQ, ybtS, ybtT, ybtU, ybtX*		*rcsA, rcsB*	*impA/tssA, sciN/tssJ, tssF, tssG, vasE/tssK, vgrG/tssI, vipA/tssB, vipB/tssC*		
214	ST 6	*fimA, fimB, fimC, fimD, fimE, fimF, fimG, fimH, fimI, fimK*	*mrkB, mrkC, mrkD, mrkF, mrkH, mrkI,*	*acrA, acrB*	*cpsACP, galF, gnd, manB, manC, ugd, wza like, wzi*	*entA, entB, entC, entE, entF, fepA, fepB, fepC, fepD, fepG, fes, ybdA, iroE, irp1, irp2, ybtA, ybtE, ybtP, ybtQ, ybtS, ybtT, ybtU, ybtX*		*rcsA, rcsB*	*impA/tssA like, sciN/tssJ, tssF, tssG, vasE/tssK, vgrG/tssI, vipA/tssB, vipB/tssC*	*glf, wbbM, wbbN, wbbO, wzm, wzt*	
217	ST 3154	*fimA, fimB, fimC, fimD, fimE, fimF, fimG, fimH, fimI, fimK*	*mrkA, mrkB, mrkC, mrkD, mrkF, mrkH, mrkI, mrkJ*	*acrA, acrB*	*cpsACP, galF, gnd, manB, manC, ugd, wza like, wzi*	*entA, entB, entC, entE, entF, fepA, fepB, fepC, fepD, fepG, fes, ybdA, iroE*		*rcsA, rcsB*	*impA/tssA like, sciN/tssJ, tssF, tssG, vasE/tssK, vgrG/tssI, vipA/tssB, vipB/tssC*	*glf, wbbM, wbbN, wbbO, wzm, wzt*	
220	ST 5382	*fimA, fimB, fimC, fimD, fimE, fimF, fimG, fimH, fimI, fimK*	*mrkA, mrkD, mrkF, mrkH, mrkI, mrkJ*	*acrA, acrB*	*cpsACP, galF, gnd, manB like, manC, ugd, wza like, wzi*	*entA, entB, entC, entE, entF, fepA, fepB, fepC, fepD, fepG, fes, ybdA, iroE,*	*allA, allB, allC, allD, allR, allS*	*rcsA, rcsB*			
234	ST 109	*fimA, fimB, fimC, fimD, fimE, fimF, fimG, fimH, fimI, fimK*	*mrkA, mrkB, mrkC, mrkD, mrkF, mrkH, mrkI, mrkJ*	*acrA, acrB*	*cpsACP, galF, gnd, manB, manC, ugd, wza like, wzi*	*entA, entB, entC, entE, entF, fepA, fepB, fepC, fepD, fepG, fes, ybdA, iroE*		*rcsA, rcsB,*	*impA/tssA, sciN/tssJ, tssF, tssG, vasE/tssK, vgrG/tssI, vipA/tssB, vipB/tssC*	*glf, wbbM, wbbN, wbbO, wzm, wzt*	
240	ST 5383	*fimC, fimD, fimF, fimG, fimH, fimI, fimK*	*mrkF, mrkH, mrkI, mrkJ*	*acrA, acrB*	*cpsACP, galF, gnd, ugd, wza like, wzi*	*entA, entB, entC, entE, entF, fepA, fepB, fepC, fepD, fepG, fes, ybdA, iroE*		*rcsA, rcsB*		*glf, wbbM, wbbN, wbbO, wzm, wzt*	
248	ST 5384		*mrkC, mrkD, mrkF, mrkH, mrkI, mrkJ*	*acrA, acrB*	*cpsACP, galF, gnd, manB, manC, ugd, wza like, wzi*	*entA, entB, entC, entE, entF, fepA, fepB, fepC, fepD, fepG, fes, ybdA, iroE*		*rcsA, rcsB*	*vasE/tssK, vgrG/tssI, vipA/tssB, vipB/tssC*	*glf, wbbM, wbbN, wbbO, wzm, wzt*	
252	ST 607	*fimA, fimB, fimC, fimD, fimE, fimF, fimG, fimH, fimI, fimK*	*mrkA, mrkB, mrkC, mrkD, mrkF, mrkH, mrkI, mrkJ*	*acrA, acrB*	*cpsACP, galF, gnd, wza like, wzi*	*entA, entB, entC, entE, entF, fepA, fepB, fepC, fepD, fepG, fes, ybdA, iroE*		*rcsA, rcsB*	*sciN/tssJ, tssF, tssG, vasE/tssK, vgrG/tssI, vipA/tssB, vipB/tssC*	*glf, wbbM, wbbN, wbbO, wzm, wzt*	
267	ST 36	*fimA, fimB, fimC, fimD, fimE, fimF, fimG, fimH, fimI, fimK*	*mrkA, mrkB, mrkC, mrkD, mrkF, mrkH, mrkI, mrkJ*	*acrA, acrB*	*cpsACP, galF, gnd, manB, manC, ugd, wza, wzi*	*entA, entB, entC, entE, entF, fepA, fepB, fepC, fepD, fepG, fes, ybdA, iroE, irp1, irp2, ybtA, ybtE, ybtP, ybtQ, ybtS, ybtT, ybtU, ybtX*		*rcsA, rcsB*	*impA/tssA, sciN/tssJ, tle1, tli1, tssF, tssG, vasE/tssK, vgrG/tssI, vipA/tssB, vipB/tssC*	*glf, wbbM, wbbN, wbbO, wzm, wzt*	
277	ST 530	*fimA, fimB, fimC, fimD, fimE, fimF, fimG, fimH, fimI, fimK*	*mrkA, mrkB, mrkC, mrkD, mrkF, mrkH, mrkI, mrkJ*	*acrA, acrB*	*cpsACP, galF, gnd, manB, manC, ugd, wza, wzi*	*entA, entB, entC, entE, entF, fepA, fepB, fepC, fepD, fepG, fes, ybdA, iroE*		*rcsA, rcsB*	*impA/tssA like, sciN/tssJ, tssF, tssG, vasE/tssK, vgrG/tssI, vipA/tssB, vipB/tssC*	*glf, wbbM, wbbN, wbbO, wzm, wzt*	
279	ST 5385	*fimA, fimB, fimC, fimD, fimE, fimF, fimG, fimH, fimI, fimK*	*mrkA, mrkB, mrkC, mrkD, mrkF, mrkH, mrkI, mrkJ*	*acrA, acrB*	*cpsACP, galF, gnd, ugd, wza, wzi*	*entA, entB, entC, entE, entF, fepA, fepB, fepC, fepD, fepG, fes, ybdA, iroE*		*rcsA, rcsB,*	*impA/tssA, sciN/tssJ, tle1, tli1, tssF, tssG, vasE/tssK, vgrG/tssI, vipA/tssB, vipB/tssC*	*wzm, wzt*	

**Table A11:** Details on the strain-specific short-read archive (SRA) accession numbers.

Sample ID	Percentage of Good Targets (SeqSphere+)	Average Coverage (Assembled) (SeqSphere+)	Approximated Genome Size (Megabases) (SeqSphere+)	Species (Kraken2)	Sequence Type	Complex Type (SeqSphere+)	SRA Accession
Iso00017	99.4	105	6.7	*Pseudomonas aeruginosa*	381	1791	SRR13617317
Iso00022	99.4	102	6.9	*Pseudomonas aeruginosa*	2483	1792	SRR13617316
Iso00032	99.2	106	6.6	*Pseudomonas aeruginosa*	3587	1793	SRR13617305
Iso00041	99.4	97	5.0	*Escherichia coli*	2 (Pasteur)	11349	SRR13617294
Iso00044	99.7	116	5.1	*Klebsiella pneumoniae*	327	5462	SRR13617283
Iso00049	98.7	94	5.2	*Escherichia coli*	3 (Pasteur)	11350	SRR13617272
Iso00060	99.6	112	5.3	*Klebsiella pneumoniae*	5379	5463	SRR13617261
Iso00068	99.6	109	4.9	*Escherichia coli*	632 (Pasteur)	11351	SRR13617250
Iso00069	99.6	104	6.8	*Pseudomonas aeruginosa*	360	1794	SRR13617239
Iso00073	99.4	104	5.8	*Klebsiella pneumoniae*	39	5464	SRR13617236
Iso00081	98.7	108	6.6	*Pseudomonas aeruginosa*	244	1795	SRR13617315
Iso00082	99.4	112	6.3	*Pseudomonas aeruginosa*	514	1796	SRR13617314
Iso00088	97.8	105	6.8	*Pseudomonas aeruginosa*	1682	1797	SRR13617313
Iso00099	99.4	106	6.6	*Pseudomonas aeruginosa*	244	1798	SRR13617312
Iso00100	99.2	108	5.5	*Klebsiella pneumoniae*	152	5465	SRR13617311
Iso00102	99.2	111	5.4	*Klebsiella pneumoniae*	514	5466	SRR13617310
Iso00106	99.5	110	6.4	*Pseudomonas aeruginosa*	1521	1799	SRR13617309
Iso00114	99.4	105	6.7	*Pseudomonas aeruginosa*	244	1800	SRR13617308
Iso00117	99.2	95	5.3	*Escherichia coli*	4 (Pasteur)	11352	SRR13617307
Iso00124	99.4	112	5.3	*Klebsiella pneumoniae*	399	5467	SRR13617306
Iso00137	99.4	110	6.4	*Pseudomonas aeruginosa*	3014	1801	SRR13617304
Iso00144	99.6	109	6.5	*Pseudomonas aeruginosa*	245	1802	SRR13617303
Iso00146	99.4	110	5.5	*Klebsiella pneumoniae*	4	5468	SRR13617302
Iso00147	99.5	108	6.6	*Pseudomonas aeruginosa*	245	1802	SRR13617301
Iso00149	99.6	104	6.9	*Pseudomonas aeruginosa*	381	1803	SRR13617300
Iso00152	99.4	98	5.2	*Escherichia coli*	22 (Pasteur)	11353	SRR13617299
Iso00153	98.5	111	6.4	*Pseudomonas aeruginosa*	704	?	SRR13617298
Iso00154	99.4	102	7.0	*Pseudomonas aeruginosa*	244	1805	SRR13617297
Iso00157	99.6	114	6.3	*Pseudomonas aeruginosa*	2616	1806	SRR13617296
Iso00160	99.2	115	6.2	*Pseudomonas aeruginosa*	170	1807	SRR13617295
Iso00162	99.1	111	6.5	*Pseudomonas aeruginosa*	274	1808	SRR13617293
Iso00176	99.0	98	5.1	*Escherichia coli*	132 (Pasteur)	11354	SRR13617292
Iso00177	99.6	108	5.5	*Klebsiella pneumoniae*	17	5469	SRR13617291
Iso00180	99.8	110	6.5	*Pseudomonas aeruginosa*	856	1809	SRR13617290
Iso00181	99.9	107	5.6	*Klebsiella pneumoniae*	5380	5470	SRR13617289
Iso00183	99.5	107	6.7	*Pseudomonas aeruginosa*	244	1795	SRR13617288
Iso00184	98.3	104	5.6	*Klebsiella variicola* subsp*. variicola*	5381	5471	SRR13617287
Iso00186	98.7	113	6.3	*Pseudomonas aeruginosa*	3588	1810	SRR13617286
Iso00190	99.7	114	6.3	*Pseudomonas aeruginosa*	871	1811	SRR13617285
Iso00195	99.5	111	6.5	*Pseudomonas aeruginosa*	988	1812	SRR13617284
Iso00196	99.5	101	7.1	*Pseudomonas aeruginosa*	2475	1813	SRR13617282
Iso00198	99.6	112	6.4	*Pseudomonas aeruginosa*	2476	1814	SRR13617281
Iso00199	99.4	108	5.6	*Klebsiella pneumoniae*	17	5472	SRR13617280
Iso00204	99.5	104	6.9	*Pseudomonas aeruginosa*	639	1815	SRR13617279
Iso00208	99.7	109	6.5	*Pseudomonas aeruginosa*	132	1816	SRR13617278
Iso00214	99.7	108	5.5	*Klebsiella pneumoniae*	6	5473	SRR13617277
Iso00217	99.8	104	5.7	*Klebsiella pneumoniae*	3154	5474	SRR13617276
Iso00218	99.7	109	6.5	*Pseudomonas aeruginosa*	856	1809	SRR13617275
Iso00220	97.8	110	5.4	*Klebsiella quasipneumoniae* subsp*. similipneumoniae*	5382	5475	SRR13617274
Iso00221	99.0	94	5.1	*Escherichia coli*	132 (Pasteur)	11354	SRR13617273
Iso00222	99.0	96	5.1	*Escherichia coli*	132 (Pasteur)	11354	SRR13617271
Iso00225	99.1	99	5.2	*Escherichia coli*	506 (Pasteur)	11355	SRR13617270
Iso00229	99.6	109	6.5	*Pseudomonas aeruginosa*	270	1817	SRR13617269
Iso00233	97.8	114	6.1	*Pseudomonas aeruginosa*	3227	1818	SRR13617268
Iso00234	99.7	111	5.5	*Klebsiella pneumoniae*	109	5476	SRR13617267
Iso00236	99.7	112	6.4	*Pseudomonas aeruginosa*	266	1819	SRR13617266
Iso00238	98.7	108	6.6	*Pseudomonas aeruginosa*	3589	1820	SRR13617265
Iso00240	98.9	112	5.4	*Klebsiella pneumoniae*	5383	5477	SRR13617264
Iso00242	98.9	111	6.4	*Pseudomonas aeruginosa*	3590	1821	SRR13617263
Iso00243	98.9	111	6.4	*Pseudomonas aeruginosa*	3590	1821	SRR13617262
Iso00245	99.3	107	4.8	*Escherichia coli*	2 (Pasteur)	11356	SRR13617260
Iso00248	97.2	108	5.5	*Klebsiella quasivariicola*	5384	5478	SRR13617259
Iso00252	99.6	112	5.3	*Klebsiella pneumoniae*	607	5479	SRR13617258
Iso00267	99.6	103	5.7	*Klebsiella pneumoniae*	36	5480	SRR13617257
Iso00270	99.2	100	4.9	*Escherichia coli*	2 (Pasteur)	11358	SRR13617256
Iso00272	99.5	109	6.5	*Pseudomonas aeruginosa*	2033	1822	SRR13617255
Iso00274	99.4	109	6.5	*Pseudomonas aeruginosa*	2033	1822	SRR13617254
Iso00277	99.4	109	5.5	*Klebsiella pneumoniae*	530	5481	SRR13617253
Iso00278	99.6	110	6.5	*Pseudomonas aeruginosa*	988	1823	SRR13617252
Iso00279	99.7	111	5.5	*Klebsiella pneumoniae*	5385	5482	SRR13617251
Iso00282	99.3	108	6.6	*Pseudomonas aeruginosa*	554	1824	SRR13617249
Iso00285	99.3	109	6.5	*Pseudomonas aeruginosa*	554	1824	SRR13617248
Iso00289	99.6	112	6.3	*Pseudomonas aeruginosa*	1485	1825	SRR13617247
Iso00290	99.7	113	6.3	*Pseudomonas aeruginosa*	1485	1825	SRR13617246
Iso00296	99.7	106	6.7	*Pseudomonas aeruginosa*	235	1826	SRR13617245
Iso00298	97.8	116	6.1	*Pseudomonas aeruginosa*	3227	1818	SRR13617244
Iso00299	99.3	108	4.6	*Escherichia coli*	1018 (Pasteur)	11357	SRR13617243
Iso00301	98.6	112	6.3	*Pseudomonas aeruginosa*	3593	1827	SRR13617242
Iso00302	99.6	113	6.3	*Pseudomonas aeruginosa*	1755	1828	SRR13617241
Iso00309	98.6	109	6.5	*Pseudomonas aeruginosa*	3592	1829	SRR13617240
Iso00310	99.3	105	6.8	*Pseudomonas aeruginosa*	532	1830	SRR13617238
Iso00312	99.4	106	6.7	*Pseudomonas aeruginosa*	381	1791	SRR13617237
